# Disaggregated Municipal Energy Consumption and Emissions in End-use Sectors in Germany and Spain for 2022

**DOI:** 10.1038/s41597-025-05938-1

**Published:** 2025-09-30

**Authors:** Shruthi Patil, Noah Pflugradt, Jann M. Weinand, Jürgen Kropp, Detlef Stolten

**Affiliations:** 1 Jülich Systems Analysis, Forschungszentrum Jülich, Wilhelm-Johnen-Straße, 52425 Jülich, NRW Germany; 2 University of Potsdam, Institute for Environmental Science and Geography, Karl-Liebknecht-Str. 24-25, Potsdam-Golm, 14476 Brandenburg, Germany; 3 Potsdam Institute for Climate Impact Research (PIK), Member of the Leibniz Association, P.O. Box 60 12 03, Potsdam, D-14412 Brandenburg, Germany; 4 Chair for Fuel Cells, RWTH Aachen University, c/o Jülich Systems Analysis, Forschungszentrum Jülich, 52425 Jülich, NRW Germany

**Keywords:** Environmental sciences, Climate sciences

## Abstract

Sectorally-detailed municipal energy consumption and emissions datasets are crucial for localized policy-making, resource allocation, and climate action planning. While some large municipalities develop bottom-up inventories, smaller ones often lack the capacity to do so. Existing studies have spatially disaggregated national totals, yet no dataset to date provides both energy consumption and emissions data across multiple sectors at the municipal level. This study addresses that gap by disaggregating national final energy consumption and emissions data in 2022 to the municipal level for Germany and Spain. The dataset covers five key end-use sectors: industry, transport, agriculture, households, and commerce. Where available, sub-sectors such as passenger and freight transport, or specific industries like chemical and paper manufacturing are further considered for disaggregation. Two main challenges are addressed: the limited availability of municipal-level proxies and the presence of missing values in proxy datasets. We apply XGBoost for imputation and implement a step-wise disaggregation using regional statistics. Using only open data, we ensure replicability, and assign confidence ratings to all values to support transparent interpretation.

## Background & Summary

Sector-specific municipal energy consumption and greenhouse gas (GHG) emissions data is a valuable resource for the energy and climate research community. For instance, the energy consumption in road transport can support the planning of electric vehicle charging infrastructure, facilitating the transition to electric mobility and assessing its impact on grid load^1^. Such granular data is also critical for formulating localized climate strategies, including Sustainable Energy and Climate Action Plans (SECAPs) led by the Covenant of Mayors^2^. These plans require comprehensive Final Energy Consumption (FEC) assessments —measuring the energy delivered to end users after conversion, transmission, and distribution losses —across key sectors including residential, Emissions Trading System (ETS) and non-ETS industries, and both public and private transport.

Few municipalities have developed their own bottom-up inventories^3,4^. When available, these inventories are often created independently, resulting in inconsistencies and data gaps across municipalities. This lack of standardisation hinders the aggregation of data to derive coherent totals at state or national levels. Furthermore, existing local inventories tend to be limited in scope; for instance, they frequently exclude cross-regional traffic, leaving a considerable share of national FEC and emissions unaccounted for in local inventories^5^. Currently, no bottom-up inventory offers comprehensive coverage of municipal-level FEC and emissions. For European Union (EU) Member States, comprehensive and official inventories are only available at the national level via Eurostat^6^.

Previous efforts to develop detailed sub-national inventories have commonly relied on spatial disaggregation techniques to construct top-down estimates. A variety of methods are documented in the literature, ranging from straightforward approaches based on area or population proportions to more sophisticated techniques employing machine learning or geostatistical models^7^. Among these, proxy-based disaggregation is particularly prevalent due to its intuitive application and reliance on domain knowledge to select appropriate spatial proxies for the data in question. This method allocates national-level values to sub-national units according to the distribution of the chosen proxy variable. For instance, national residential building emissions can be disaggregated to the municipal level using population shares, given that population density is a key driver of residential energy use and associated emissions.

Employing a proxy data-based approach, the Emissions Database for Global Atmospheric Research (EDGAR)^8^ provides GHG emissions from various sectors, including power generation, industry, residential activities, and transport, at the level of federal states globally. The federal state level, a coarser spatial resolution than municipalities, is chosen here due to the lack of relevant proxy datasets available at the municipal level across the globe. The work aimed to strike a balance between the accuracy and the granularity of the created data.

Municipal-scale disaggregation of sectoral emissions in Europe has been attempted in a previous study^9^, but relies on a single data source, OpenStreetMap^10^, as the primary spatial proxy. While this approach incorporates features such as fuel stations and buildings, it overlooks other potentially influential factors that could enhance accuracy. For example, incorporating heating degree days could better account for variations in energy demand, and thus emissions, across different climatic regions. Similarly, using data on vehicle fleets or traffic volumes, rather than the mere presence of fuel stations, would provide a more representative proxy for local road transport emissions. Other studies have also undertaken spatial disaggregation of emissions, but they are typically limited in scope —focusing on a specific sector and/or operating at coarser spatial resolutions^7^.

Regarding the disaggregation of FEC, the Hotmaps project^11^ offers residential and non-residential FEC data at a high spatial resolution of 1 hectare across Europe. This granularity enables alignment with municipal boundaries to derive municipal-level estimates. However, to the best of the authors’ knowledge, no existing inventory currently provides both FEC and emissions data with detailed sectoral resolution at the municipal scale.

To address this data gap, the present study develops sectorally detailed, municipal-level inventories of FEC and GHG emissions for the year 2022. This is achieved by spatially disaggregating national-level data sourced from Eurostat to the municipal scale. The analysis encompasses five principal end-use sectors: industry, transport, agriculture, households, and commerce. Where Eurostat provides additional sectoral granularity —such as within the transport sector (e.g., rail, passenger cars, motorcycles) or specific industrial sub-sectors (e.g., iron and steel, non-ferrous metals, textile and leather) —these disaggregated categories are retained to enhance the level of detail. The resulting inventories are thus both spatially and sectorally resolved, offering a comprehensive basis for local-level energy and climate analysis.

The key innovation of this work lies in addressing the limited availability of relevant spatial proxies at the municipal level. To overcome this challenge, a step-wise disaggregation approach is adopted. First, proxies available at intermediate spatial scales, such as districts or provinces, are disaggregated to the municipal level. These refined proxies are then used to disaggregate national FEC and emissions data. Beyond spatial resolution limitations, many proxy datasets also suffer from missing values. To address this, a machine learning model, specifically XGBoost^12^, is trained to impute missing data prior to disaggregation, thereby improving the robustness and completeness of the resulting inventories. Importantly, all proxy datasets used in this study are sourced from publicly available databases, enabling replication of the methodology and facilitating its application to countries beyond those examined here.

This study focuses on Germany and Spain. Germany is selected as it is the largest GHG emitter in the EU^13^, while Spain is included due to its national climate policies that emphasize the critical role of local governments in achieving climate targets, as outlined in its National Energy and Climate Plan^14^. In this context, the development of top-down, spatially disaggregated FEC and emissions inventories can support the Spanish national government in tailoring policies to local needs, and assist municipal authorities in designing climate action plans that are aligned with national strategies.

By providing detailed, spatially disaggregated energy and emissions data, this work offers municipalities a valuable resource for meeting inventory preparation and reporting requirements. These datasets are particularly beneficial for local governments with limited statistical infrastructure or technical capacity to compile sector-specific baseline inventories. Moreover, because the generated inventories are methodologically consistent across municipalities, they enable meaningful comparisons that can inform localized policy-making, resource allocation, and the alignment of municipal strategies with national climate goals.

## Methods

**Fig. 1:**
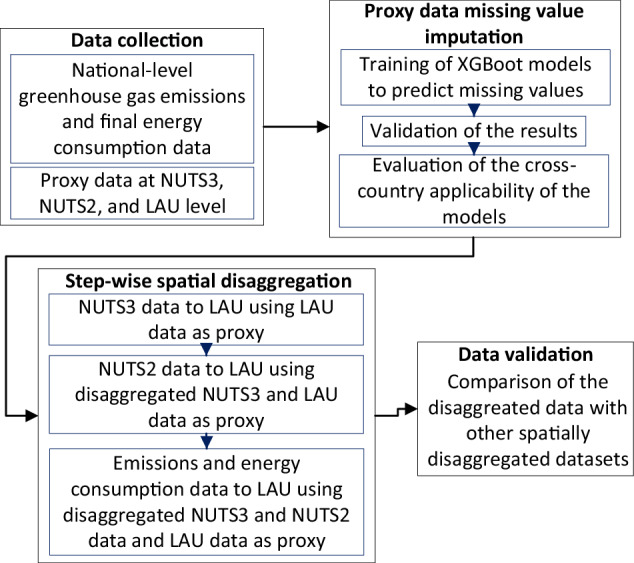
The steps involved in the spatial disaggregation of emissions and Final Energy Consumption (FEC) data from country (NUTS0) to municipal (LAU) level.

The spatial disaggregation of FEC and emissions data is carried out in four steps, as illustrated in Fig. 1. The following sub-sections describe each step in detail.

### Data collection

For the disaggregation work, this study utilizes three types of data: (i) FEC data for various sub-sectors (ii) emissions data for various sub-sectors and (iii) proxy data relevant to each sub-sector, which facilitates the disaggregation of FEC and emissions data.

**Fig. 2:**
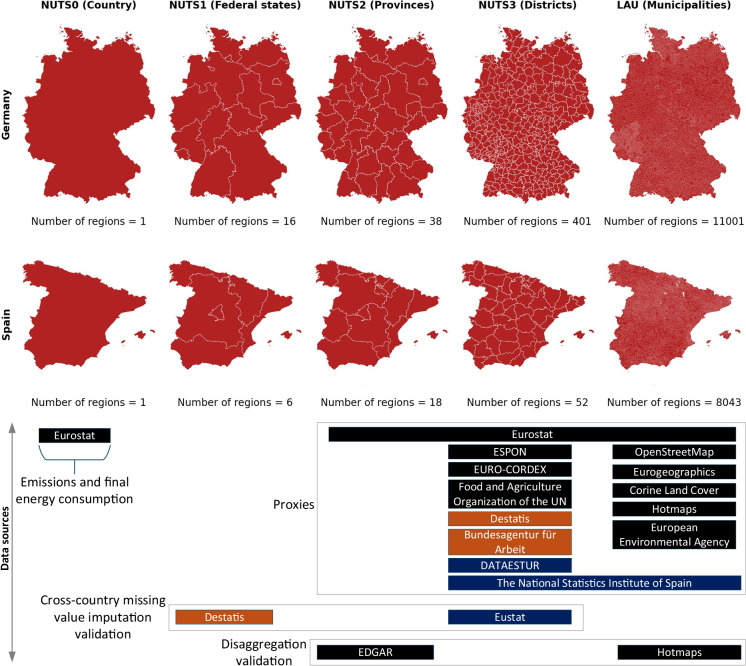
The spatial hierarchy in Germany and Spain, showing the availability of various proxy datasets from public data sources at different spatial levels. The data sources highlighted in orange and blue provide data only for Germany and Spian, respectively. Proxy data undergoes a step-wise spatial disaggregation to achieve final proxies at the LAU level. Emissions and FEC data, available at the NUTS0 level from Eurostat, is then disaggregated to LAU based on these final proxies.

FEC and emissions data are collected at the national level, whereas proxy datasets are collected at various sub-national spatial resolutions. Figure 2 illustrates the Nomenclature of Territorial Units for Statistics (NUTS) spatial hierarchy in Germany and Spain. Beginning at the national level (NUTS0), the hierarchy increases in spatial resolution through successive subdivisions: NUTS1 (federal states), NUTS2 (provinces), and NUTS3 (districts). Below NUTS3, municipalities —referred to as Local Administrative Units (LAUs) —represent the most granular level of administrative geography in both countries. The size of LAUs varies significantly. In Germany, the smallest LAU is Insel Lütje Hörn, covering just 0.096 *km*^2^, while the largest is Berlin at 891.80 *km*^2^. In Spain, Emperador is the smallest LAU (0.026 *km*^2^) and Cáceres the largest (1,750.26 *km*^2^).

The details regarding the collection of each dataset are discussed in the following sections.

#### Final energy consumption

**Fig. 3:**
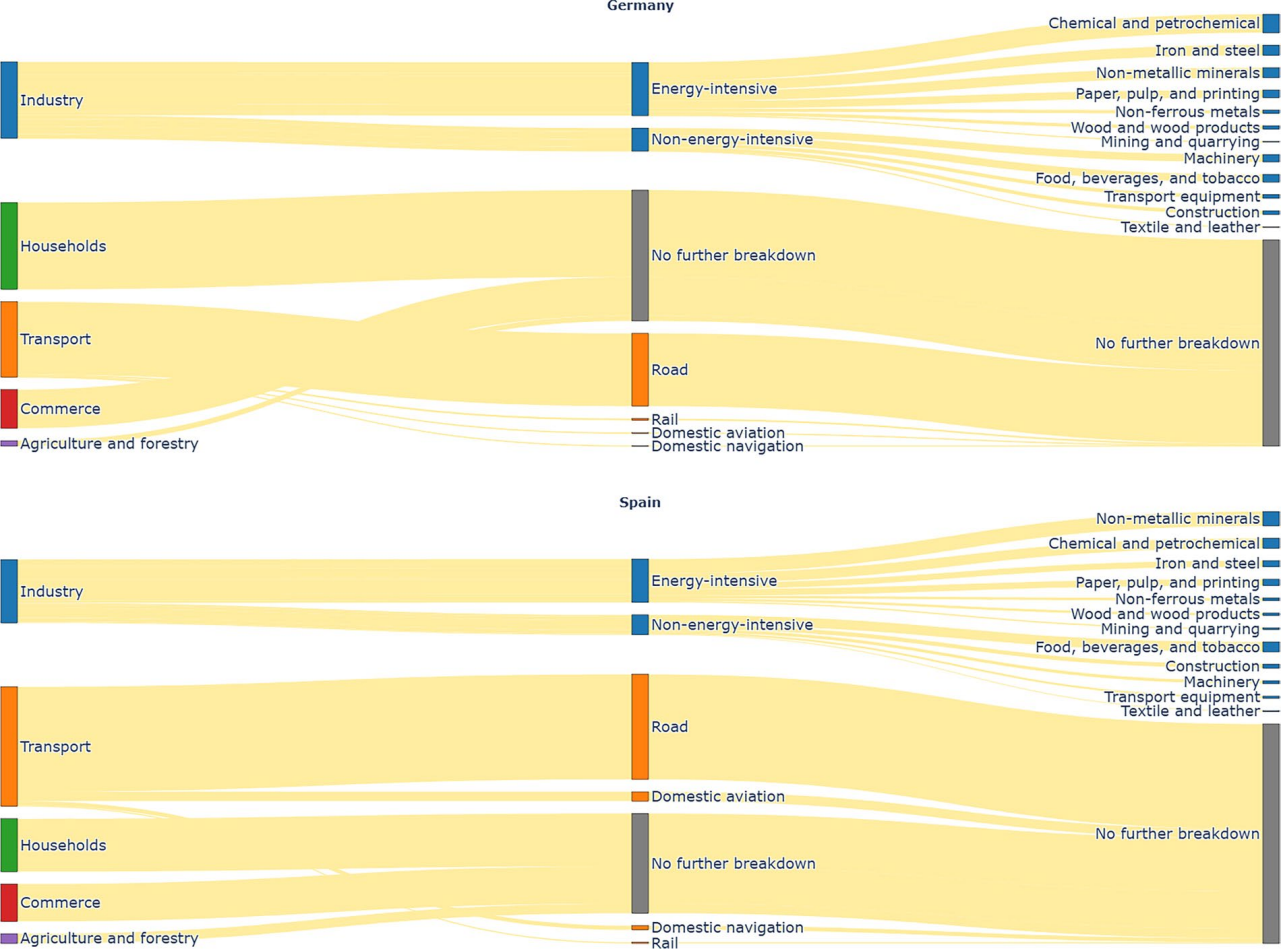
Breakdown of end-use FEC sectors as reported in Eurostat, with Germany at the top and Spain at the bottom.

The FEC data, reported at the national level, is imported from the energy balance sheet published on Eurostat^15^, for the year 2022. While the FEC data for both energy and non-energy use is reported on Eurostat, only the energy use FEC is considered here. The breakdown of the end-use sectors for emissions reporting on Eurostat is shown in Fig. 3.

The industry sector is broken down into energy-intensive and non-energy-intensive industries. Energy-intensive industries include iron and steel, chemical and petrochemical, non-ferrous metals, non-metallic minerals, mining and quarrying, paper, pulp, and printing, and wood and wood products manufacturing industries^16^. Non-energy-intensive industries include transport equipment, machinery, food, beverages, and tobacco, textile and leather manufacturing industries, construction, and other industries that are not specified elsewhere^16^. A similar categorisation is provided by Eurostat.

The transport sector is categorized into rail, road, domestic aviation, and domestic navigation. Additionally, the commerce and agriculture and forestry sectors are included, though they are not further broken down.

#### Greenhouse gas emissions

**Fig. 4:**
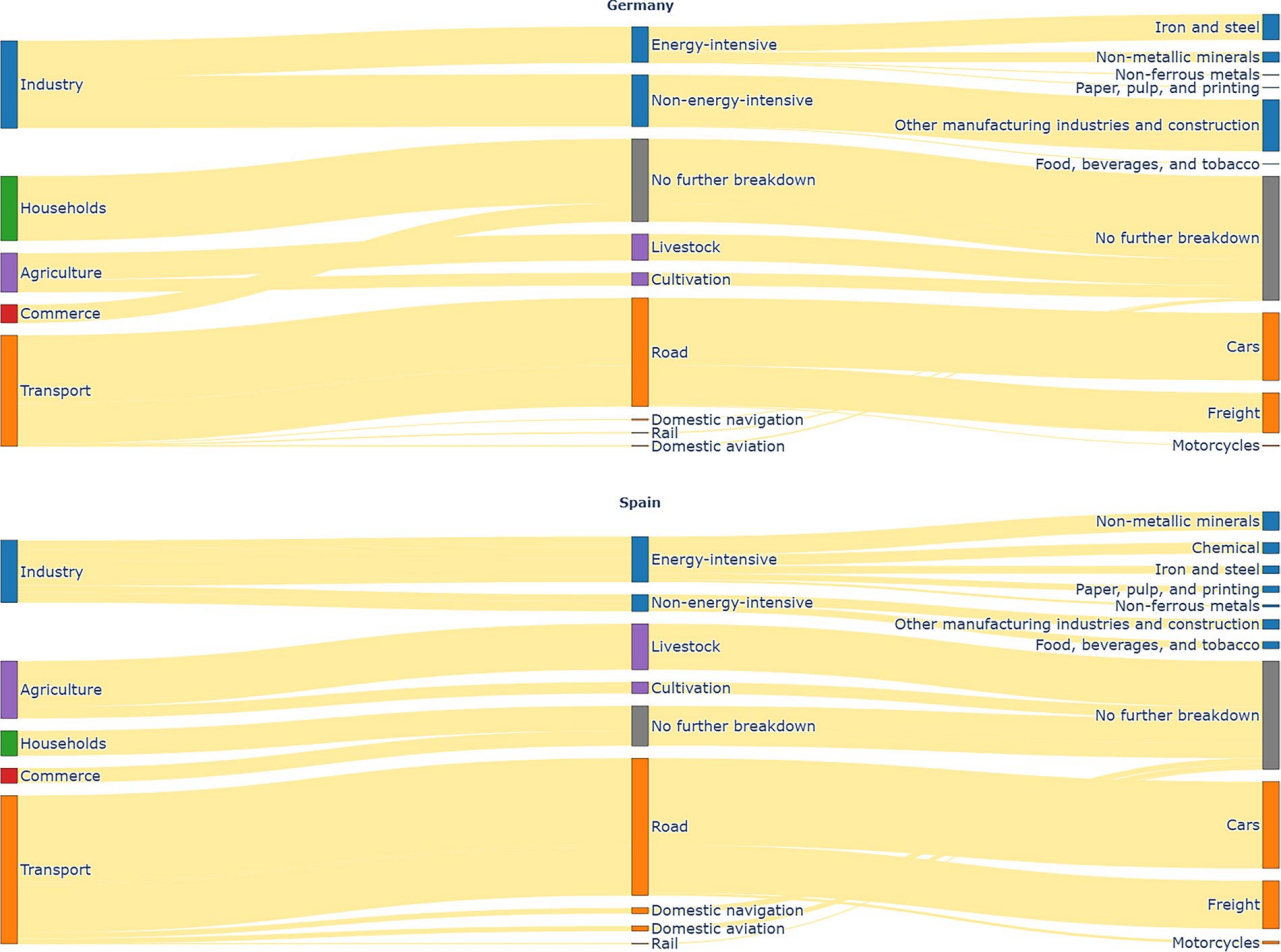
Breakdown of end-use emission sectors as reported in Eurostat, with Germany at the top and Spain at the bottom. Note: Emissions from the chemical industry are not reported for Germany on Eurostat and are therefore absent from the figure. Consequently, emissions from energy-intensive industries appear lower than those from non-energy-intensive industries.

The GHG emissions data used in this study is sourced from Eurostat^17^, for the year 2022. Figure 4 illustrates how end-use sectors are categorized for emissions reporting by Eurostat. Compared to the FEC sector classification, the industry sector emissions data provided by Eurostat is less detailed. Notably, emissions from the chemical industry are not reported for Germany, and as such, these emissions are not disaggregated in this study.

In contrast, the transport sector is more granular in the Eurostat data compared to the FEC categorisation. Here, sub-sectors such as rail, road, domestic aviation, and domestic navigation, are further broken down into categories like light-duty trucks, heavy-duty trucks and buses, cars, and motorcycles. For the purposes of this study, emissions from light-duty trucks and heavy-duty trucks and buses are grouped under freight transport, as specific proxies for these vehicle types are not available in publicly accessible datasets.

It is important to highlight that GHG emissions in the sectors examined here primarily stem from fuel combustion. Process-related emissions in the industrial sector are excluded, with one exception: the agriculture sector, which includes non-combustion emissions. Agricultural emissions are considered under two categories-livestock and cultivation. Although Eurostat provides further disaggregation into subcategories such as enteric fermentation, manure management, agricultural soil management, and crop residue burning, only livestock and cultivation are included in this study due to the absence of matching proxies in open-source data.

#### Proxy data

Proxy data serves as the foundation for spatially disaggregating FEC and emissions across different end-use sectors. Unlike the FEC and emissions data, which is collected for the year 2022, proxy data spans multiple years, as certain datasets —such as land use and land cover —are not updated annually. However, all proxy datasets used represent the most recent year for which data was available at the time of collection.

The selection of proxy data was guided by the following criteria:

- **Availability in open databases:** The data must be accessible through publicly available databases to enhance transparency and reproducibility.
- **Sub-national resolution:** The data should be available at a sub-national scale, such as NUTS1, NUTS2, NUTS3, or LAU, to facilitate the disaggregation of emissions and FEC data reported at NUTS0.
- **Relevance to end-use sectors:** The selected data must be relevant to at least one of the end-use sectors analyzed in this study. For instance, population data can be used to disaggregate household-related emissions and FEC. Heating degree days help refine the spatial distribution of FEC by accounting for higher heat demand in colder regions. In addition, industrial locations and employment data provide insights into the spatial distribution of industrial emissions and FEC. Furthermore, vehicle stock data supports the disaggregation of road transport emissions and energy consumption. Finally, land use and land cover classifications (e.g., rice fields, vineyards) assist in distributing cultivation-related FEC and emissions.
- **Data completeness:** The dataset should have minimal missing values to ensure a robust analysis. Here, less than 20% missing data is preferred.

The proxies are obtained from various publicly available databases, including Eurostat, Corine Land Cover^18^ and OpenStreetMap. A comprehensive overview of all data sources is provided in Fig. 2. The following sub-sections provide an overview of the collected proxy data at different spatial levels, beginning with the LAU level.

**Proxy data at LAU level**. The datasets collected at the LAU level for Germany and Spain are summarized in Tables 1 and 2. These include general demographic and geographic statistics, such as population and area, sourced from Eurostat, which are directly available for each LAU region. However, not all relevant datasets are directly available at the LAU level. In such cases, fine-scale gridded or vector datasets are spatially overlaid with LAU geometries to derive region-specific aggregates.

**Table 1:** LAU-level proxy data collected from Eurostat, Hotmaps, European Environmental Agency, and The National Statistics Institute of Spain.

Coverage	Data source	Variable	Unit
Germany and Spain	Eurostat^31^	Population	number
		Area	square kilometer
	Hotmaps^32^	Number of iron and steel industries	number
		Number of cement industries	number
		Number of refineries	number
		Number of paper and printing industries	number
		Number of chemical industries	number
		Number of glass industries	number
		Number of non-ferrous metals industries	number
		Number of non-metallic minerals industries	number
	European Environmental Agency^19^	Average air pollution due to PM2.5	ug/m3
		Average air pollution due to NO2	ug/m3
		Average air pollution due to O3	ug/m3
		Average air pollution due to PM10	ug/m3
Spain	The National Statistics Institute of Spain^23^	Utilized agricultural area	square kilometer

**Table 2:** LAU-level proxy data collected from Corine Land Cover, Eurogeographics, and OpenStreetMap.

Data source	Variable	Unit
Corine Land Cover^18^	Continuous urban fabric cover	square kilometer
	Discontinuous urban fabric cover	square kilometer
	Industrial or commercial units cover	square kilometer
	Port areas cover	square kilometer
	Airports cover	square kilometer
	Mineral extraction sites cover	square kilometer
	Dump sites cover	square kilometer
	Construction sites cover	square kilometer
	Green urban areas cover	square kilometer
	Sport and leisure facilities cover	square kilometer
	Non irrigated arable land cover	square kilometer
	Permanently irrigated land cover	square kilometer
	Rice fields cover	square kilometer
	Vineyards cover	square kilometer
	Fruit trees and berry plantations cover	square kilometer
	Olive groves cover	square kilometer
	Pastures cover	square kilometer
	Permanent crops cover	square kilometer
	Complex cultivation patterns cover	square kilometer
	Agriculture with natural vegetation cover	square kilometer
	Agroforestry areas cover	square kilometer
	Broad leaved forest cover	square kilometer
	Coniferous forest cover	square kilometer
	Mixed forest cover	square kilometer
	Natural grasslands cover	square kilometer
	Moors and heathland cover	square kilometer
	Sclerophyllous vegetation cover	square kilometer
	Transitional woodland shrub cover	square kilometer
	Beaches dunes and sand cover	square kilometer
	Bare rocks cover	square kilometer
	Sparsely vegetated areas cover	square kilometer
	Burnt areas cover	square kilometer
	Glaciers and perpetual snow cover	square kilometer
	Inland marshes cover	square kilometer
	Peat bogs cover	square kilometer
	Salt marshes cover	square kilometer
	Salines cover	square kilometer
	Intertidal flats cover	square kilometer
	Water courses cover	square kilometer
	Water bodies cover	square kilometer
	Coastal lagoons cover	square kilometer
	Estuaries cover	square kilometer
	Sea and ocean cover	square kilometer
Eurogeographics^33^	Railway network	kilometer
OpenStreetMap^10^	Road network	kilometer
	Number of buildings	number

All the variables are available for both Germany and Spain.

For example, land use and land cover information, including classes such as continuous urban fabric, is obtained from the Corine Land Cover database. This source provides raster data at a spatial resolution of 100 square meters, which allows for accurate spatial aggregation of land cover types within each LAU boundary. Similarly, air pollution data is sourced from the European Environment Agency^19^, available as gridded data at a resolution of 1 square kilometer.

In addition to raster sources, vector datasets are also used. The railway network data is obtained from EuroGeographics, while OpenStreetMap provides detailed information on road networks and building counts. These vector datasets are intersected with LAU geometries to extract relevant spatial indicators for each municipality.

Data on industrial sites was available in three databases: sEEnergies^20^, Global Steel Plant Tracker^21^, and Hotmaps. To determine the most suitable source, the datasets were compared for their level of detail and coverage.

- **sEEnergies** provides industrial site locations along with fuel and electricity demand information for industries such as iron and steel, chemicals, non-ferrous metals, non-metallic minerals, paper and printing, and refineries.
- **Global Steel Plant Tracker** focuses solely on iron and steel plant locations, annotating them with energy demand and employment data, though many sites lack complete information.
- **Hotmaps** includes locations for cement and glass industries in addition to those covered by sEEnergies, with emissions data provided for each site, though data is missing for many locations.

**Fig. 5:**
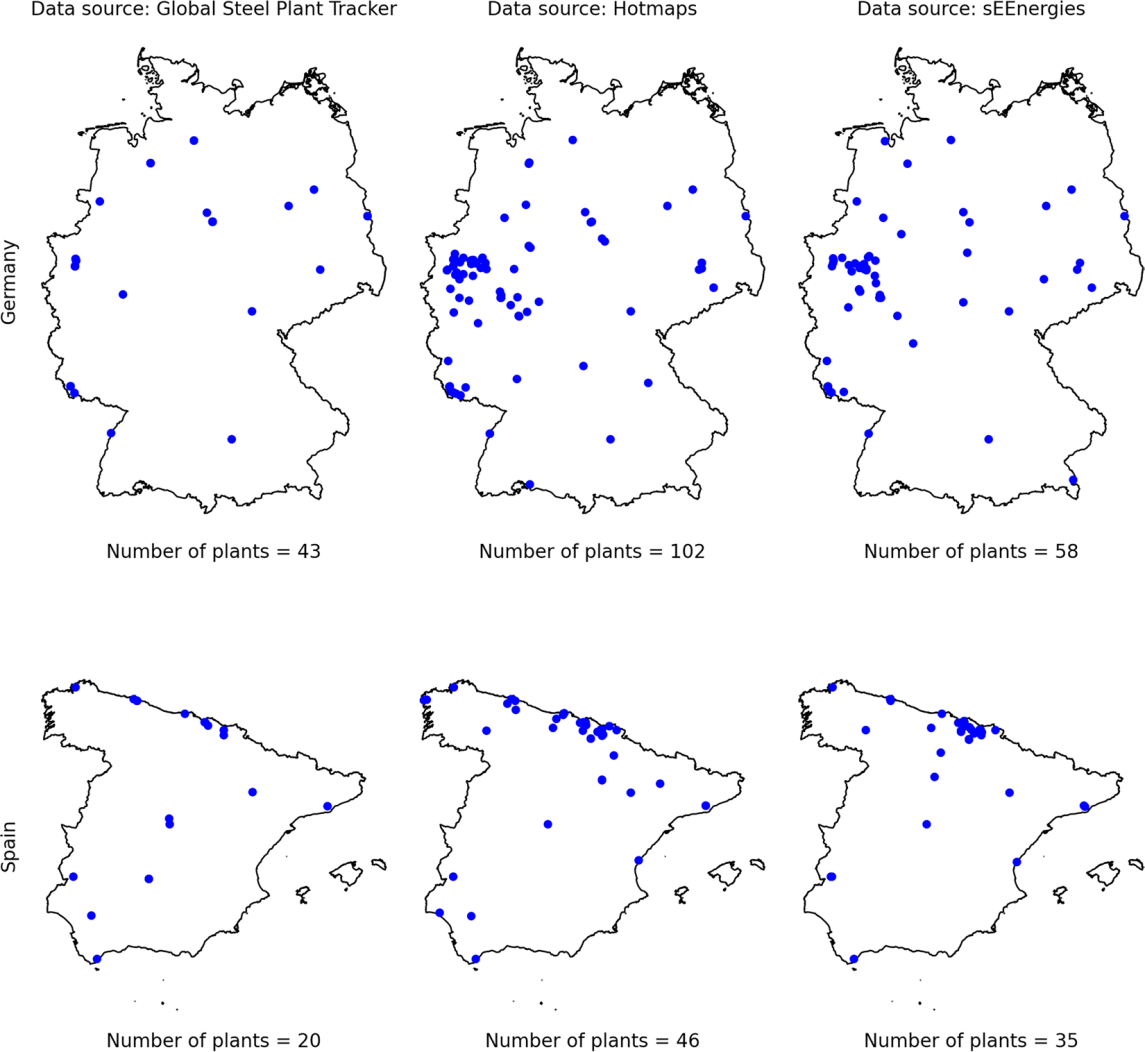
The distribution and number of iron and steel industries as reported by three open databases: Global Steel Plant Tracker, Hotmaps, and sEEnergies. The figure highlights the differences in coverage among these sources, with Hotmaps providing the most comprehensive dataset.

A comparative analysis was performed to select the most comprehensive source for each sector. For the iron and steel industry, a comparison of site counts across the three datasets was conducted, as illustrated in Fig. 5. For other industries, a comparison between sEEnergies and Hotmaps was performed (see Table 3). Hotmaps reports a higher number of industrial sites across most categories, except for paper and printing industries in Germany, where sEEnergies provides higher counts. Based on this analysis, the Hotmaps database was selected as the primary source for obtaining LAU-level industry data, ensuring comprehensive coverage and consistency across sub-sectors.

**Table 3:** Number of different industries as reported by Hotmaps and sEEnergies open databases.

Industry	Hotmaps		sEEnergies	
	Germany	Spain	Germany	Spain
Chemical industries	118	25	44	8
Non-ferrous metals industries	40	4	17	3
Non-metallic minerals industries	160	77	289	52
Paper and printing industries	140	159	65	59
Refineries	26	19	9	8

**Proxy data at NUTS3 level**. Table 4 provides an overview of the data collected for the German and Spanish NUTS3 regions. Basic statistical information, such as employment and gross domestic product, is published by Eurostat at this spatial level. Heating and cooling degree days and livestock population datasets are available in raster format. With a resolution of approximately 10 square kilometers, these datasets align well with NUTS3 regions, enabling spatial overlap and aggregation at the NUTS3 level. Additionally, some datasets are available only in one of the two countries —for example, sectorally detailed employment data from the Federal Employment Agency^22^ in Germany and company counts from the National Statistics Institute of Spain^23^.

**Table 4:** NUTS3-level proxy data collected from different data sources.

Coverage	Data source	Variable	Unit
Germany and Spain	Eurostat	Gross domestic product^34^	million Euros
		Road transport of freight^35^	Mt
		Employment in manufacturing^36^	number
		Employment in construction^36^	number
		Employment in agriculture, forestry and fishing^36^	number
	ESPON^37^	Soil sealing	square kilometer
	EURO-CORDEX^38^	Heating degree days	heating degree days
		Cooling degree days	cooling degree days
	Food and Agriculture Organization of the UN^39^	Number of buffaloes	number
		Number of cattle	number
		Number of pigs	number
		Number of sheeps	number
		Number of chickens	number
		Number of goats	number
Germany	Bundesagentur für Arbeit^40^	Employment in textile and leather manufacturing	number
		Employment in food and beverage manufacturing	number
		Employment in mechanical and automotive engineering	number
		Employment in mechatronics, energy and electrical	number
		Employment in wood processing	number
	Destatis^24^	Number of passenger cars emission group euro 1	number
		Number of passenger cars emission group euro 2	number
		Number of passenger cars emission group euro 3	number
		Number of passenger cars emission group euro 4	number
		Number of passenger cars emission group euro 5	number
		Number of passenger cars emission group euro 6r	number
		Number of passenger cars emission group euro 6dt	number
		Number of passenger cars emission group euro 6d	number
		Number of passenger cars emission group euro other	number
		Residential building living area	square kilometer
		Non-residential building living area	square kilometer
Spain	The National Statistics Institute of Spain^23^	Number of commercial and service companies	number
	DATAESTUR^41^	Average daily traffic - light duty vehicles	number

**Proxy data at NUTS2 level**. Table 5 summarizes the data available for the German and Spanish NUTS2 regions. All the datasets collected at this level are sourced from Eurostat.

**Table 5:** NUTS2-level proxy data collected from Eurostat.

Data source	Variable	Unit
Eurostat	Number of motorcycles^42^	number
	Air transport of passengers^43^	number
	Air transport of freight^44^	Mt

All the variables are available for both Germany and Spain.

### Missing value imputation

The collected proxy datasets exhibit missing values. Imputing these values is a critical step in spatial disaggregation workflows, as complete proxy data is essential for distributing national totals. Table 6 provides an overview of the number and percentage of missing values identified in the collected proxy data. These gaps are primarily found in datasets that are available only for either Germany or Spain, often due to strict data protection regulations preventing certain regions from reporting data. Consequently, missing values must be imputed using relevant statistical indicators, such as land use and land cover data when estimating the utilized agricultural area.

**Table 6:** Number of missing values per variable with missing values.

Spatial level	Variable	Number of missing values	
		(Percentage of missing values)	
		Germany	Spain
LAU	Utilized agricultural area	—	348 (4.33%)
NUTS3	Number of commerical and service companies	—	3 (5.77%)
	Average daily traffic - light duty vehicles	—	10 (19.23%)
	Employment in textile and leather manufacturing	34 (8.48%)	—
	Employment in food and beverage manufacturing	2 (0.5%)	—
	Employment in mechanical and automotive engineering	1 (0.25%)	—
	Employment in mechatronics, energy and electrical	1 (0.25%)	—
	Employment in wood processing	2 (0.5%)	—
	Number of passenger cars emission group euro 1	2 (0.50%)	—
	Number of passenger cars emission group euro 2	2 (0.50%)	—
	Number of passenger cars emission group euro 3	2 (0.50%)	—
	Number of passenger cars emission group euro 4	2 (0.50%)	—
	Number of passenger cars emission group euro 5	2 (0.50%)	—
	Number of passenger cars emission group euro 6r	2 (0.50%)	—
	Number of passenger cars emission group euro 6dt	2 (0.50%)	—
	Number of passenger cars emission group euro 6d	2 (0.50%)	—
	Number of passenger cars emission group euro other	2 (0.50%)	—
	Residential building living area	1 (0.25%)	—
	Non-residential building living area	1 (0.25%)	—

Note: The number of data records at LAU-level in Germany and Spain are 11087 and 8043, respectively. The number of data records at NUTS3-level in Germany and Spain are 401 and 52, respectively.

To impute these missing values XGBoost model is employed. Since the spatial distribution process is relative, data quality in one region directly influences the accuracy of all others. Therefore, it is crucial to assess the model’s performance. To this end, we conduct two evaluations: (1) assessing the predictive accuracy within a country by setting aside a portion of the data for validation, and (2) evaluating the model’s predictive capacity in a country where the dataset is entirely absent. The latter is achieved by leveraging available data at intermediate spatial levels, such as states, within the country. The data sources employed in this cross-country missing value imputation evaluation are listed in Fig. 2. The training and validation of the model, as well as the evaluation of the missing value prediction across countries is explained in the following.

**XGBoost model training**. The datasets with missing values at the LAU level are imputed by training an XGBoost model using all other LAU-level variables as potential predictors. Before selecting the final predictors, two preprocessing steps are performed to eliminate certain variables:

- **Removal of non-informative predictors:** Any predictors that have the same value across all regions are discarded because they lack predictive capability.
- **Correlation analysis:** A pairwise correlation among all potential predictors is examined. If two variables exhibit an absolute correlation of 0.9 or higher, only one is retained to prevent over-representation of highly similar variables in the model.

**Fig. 6:**
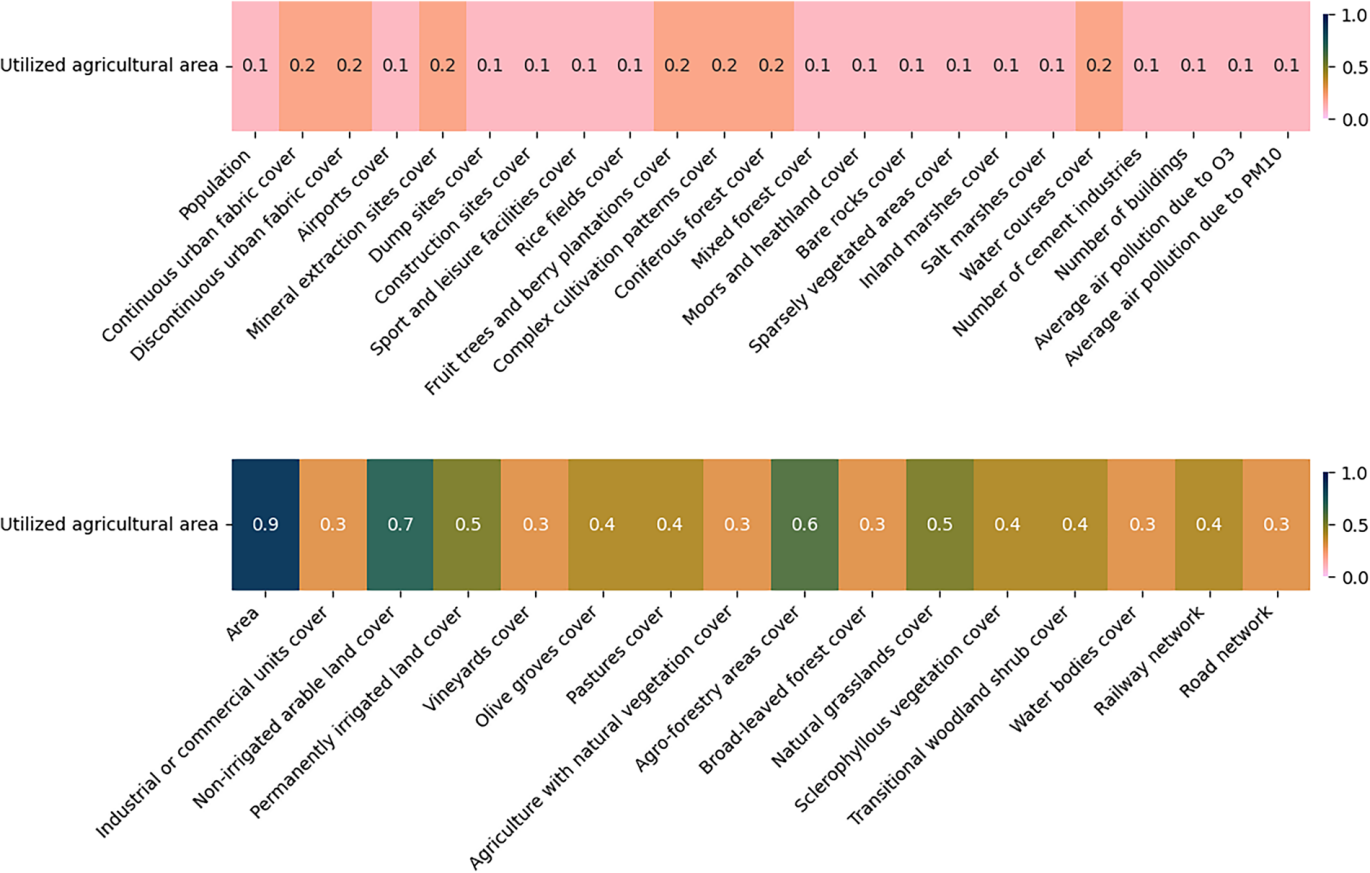
The absolute correlations between utilized agricultural area and different predictors at LAU level. The figure is divided into two sections: the top half displays the least correlated variables, while the bottom half highlights the most correlated ones. For imputing missing values in utilized agricultural area, predictors with correlations of at least 0.1 are used in one set of experiments, while those with correlations of at least 0.5 are considered in another.

The final dataset used as input for the XGBoost model consists of the selected predictors and the variable to be imputed, with only complete records included. Prior to training, 10% of the data is reserved for model validation, while the remaining 90% is utilized for two experimental setups. In the first setup, predictors with an absolute Pearson correlation of at least 0.1 with the variable to be imputed are included. In the second setup, the correlation threshold is increased to 0.5. Figure 6 presents the correlations between “utilized agricultural area” and the various predictors used.

In both sets of experiments, hyperparameter tuning is performed using a grid search on the XGBoost model. The hyperparameters tuned include *n*_*estimators*, *learning*_*rate*, and *max*_*depth*, with the model optimized for minimal Root Mean Squared Error (RMSE). To calculate the RMSE, 5-fold cross-validation is applied on the training data, splitting it into five folds. The RMSE is computed for each fold by training the model on four folds and validating on the remaining fold, and the average RMSE across all folds is used as the performance metric for hyperparameter optimization. The final model for data imputation is the one with hyperparameter combination that yields the lowest RMSE.

**Fig. 7:**
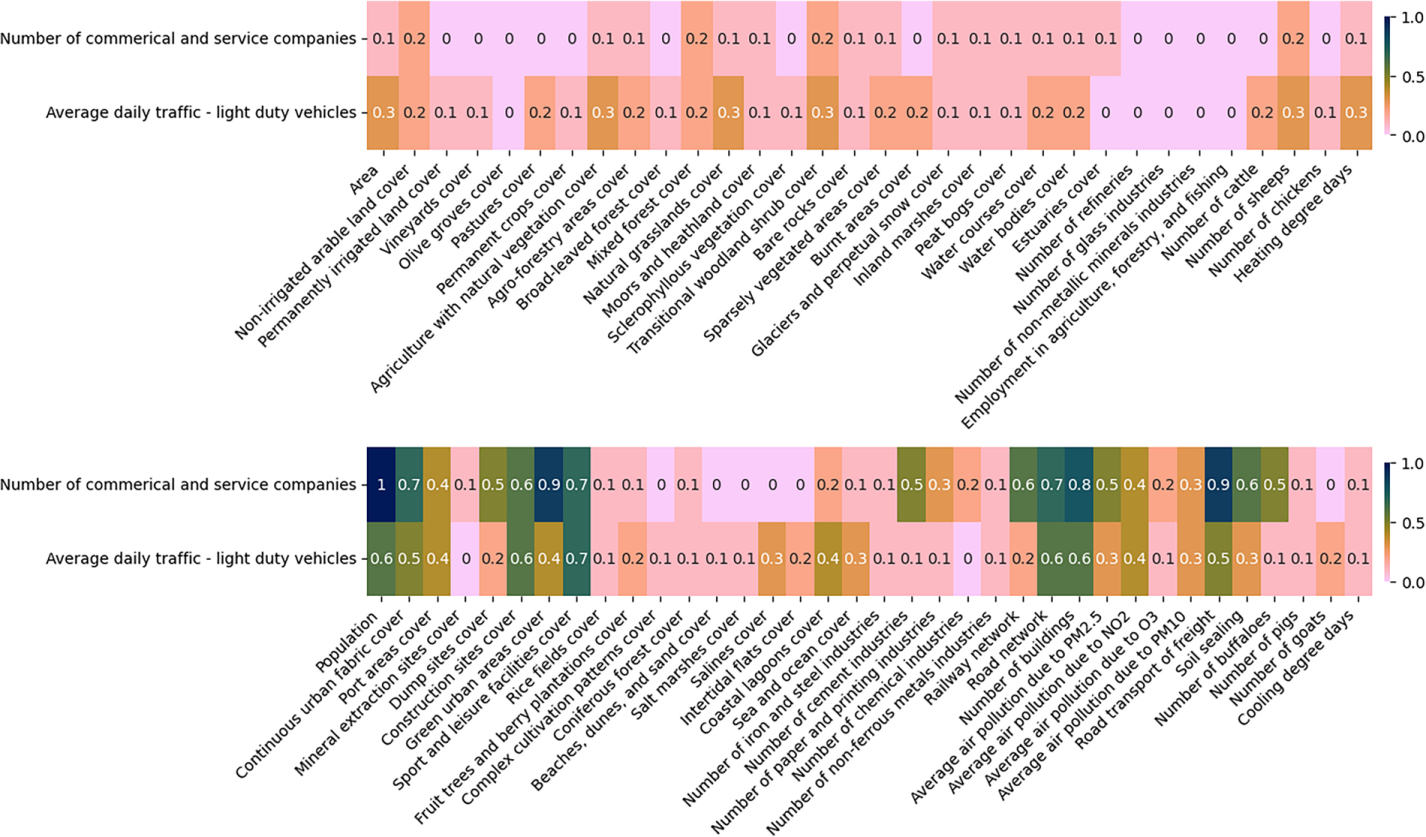
The absolute correlations between number of commercial and service companies and average daily traffic by light duty vehicles, and different predictors at NUTS3 level. The figure is divided into two sections: the top half displays the least correlated variables, while the bottom half highlights the most correlated ones. For imputing missing values, predictors with correlations of at least 0.1 are used in one set of experiments, while those with correlations of at least 0.5 are considered in another.

**Fig. 8:**
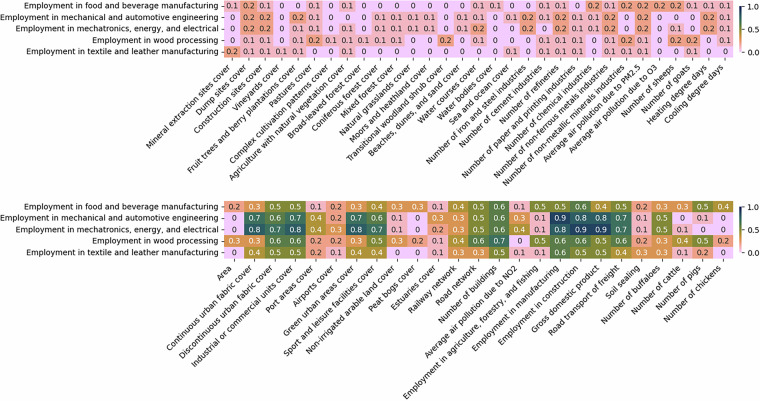
The absolute correlations between employment data and different predictors at NUTS3 level. The figure is divided into two sections: the top half displays the least correlated variables, while the bottom half highlights the most correlated ones. For imputing missing values, predictors with correlations of at least 0.1 are used in one set of experiments, while those with correlations of at least 0.5 are considered in another.

**Fig. 9:**
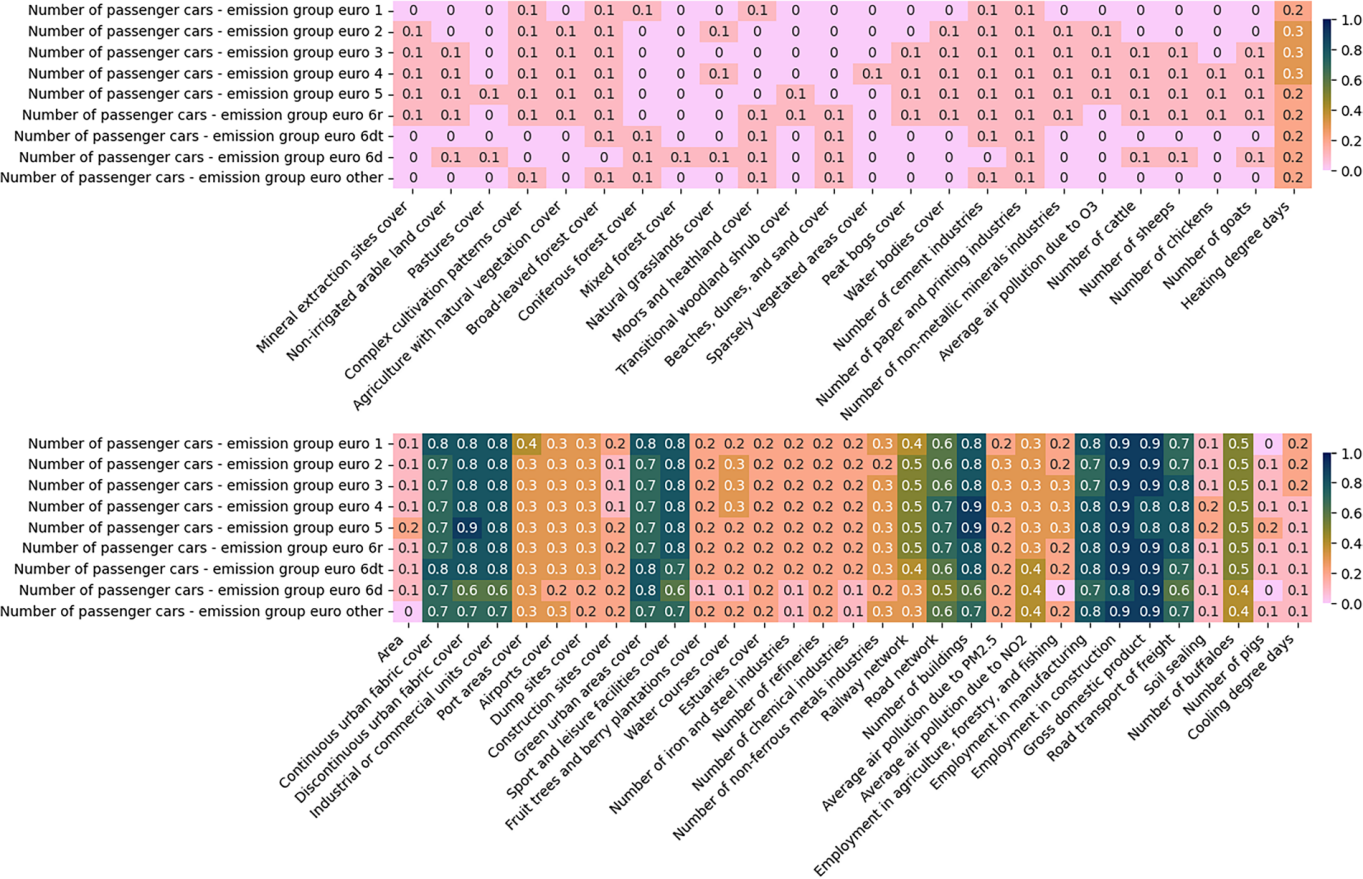
The absolute correlations between the number of passenger cars per emission group and different predictors at NUTS3 level. The figure is divided into two sections: the top half displays the least correlated variables, while the bottom half highlights the most correlated ones. For imputing missing values, predictors with correlations of at least 0.1 are used in one set of experiments, while those with correlations of at least 0.5 are considered in another.

**Fig. 10:**
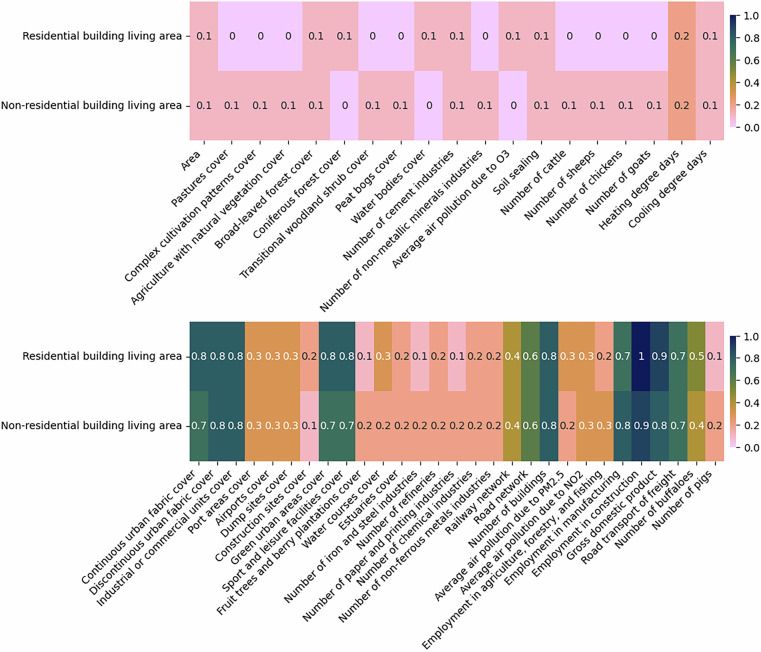
The absolute correlations between the building living area and different predictors at NUTS3 level. The figure is divided into two sections: the top half displays the least correlated variables, while the bottom half highlights the most correlated ones. For imputing missing values, predictors with correlations of at least 0.1 are used in one set of experiments, while those with correlations of at least 0.5 are considered in another.

A similar approach is used in the case of variables with missing values at the NUTS3 level. Here, the potential predictors are all variables at NUTS3 level without missing values, as well as LAU variables with no missing data, aggregated to the NUTS3 level. Figures 7, 8, 9, and 10 illustrate the correlations between the NUTS3 variables with missing values and various predictors.

**XGBoost model validation**. Table 7 presents the training and validation errors corresponding to the previously discussed correlation thresholds. While a lower RMSE indicates better model performance, its lack of fixed upper or lower bounds makes accuracy interpretation challenging. Therefore, the R-squared error is also provided, where values closer to 1 signify better performance.

**Table 7:** RMSE and R-squared scores on the training and validation datasets when using the XGBoost model for missing value imputation.

Variable	Correlation threshold ≥ 0.1				Correlation threshold ≥ 0.5			
	Training		Validation		Training		Validation	
	RMSE	R2	RMSE	R2	RMSE	R2	RMSE	R2
Utilized agricultural area	20.31	0.87	20.01	0.89	22.01	0.85	23.28	0.85
Number of commerical and service companies	49877.05	0.58	27353.46	0.61	47208.70	0.66	30192.07	0.53
Average daily traffic - light duty vehicles	689.45	0.18	719.52	−0.45	847.75	−0.89	973.89	−1.67
Employment in textile and leather manufacturing	290.16	0.27	529.56	0.10	311.57	0.14	505.69	0.18
Employment in food and beverage manufacturing	737.97	0.22	1482.62	0.29	720.42	0.29	1724.07	0.04
Employment in mechanical and automotive engineering	2083.65	0.79	1208.13	0.91	1764.42	0.83	1110.13	0.92
Employment in mechatronics, energy and electrical	1148.96	0.84	1023.46	0.82	1028.12	0.87	830.13	0.88
Employment in wood processing	427.62	0.51	259.76	0.51	464.37	248.52	376.86	0.02
Number of passenger cars emission group euro 1	378.22	0.84	155.81	0.92	355.74	0.84	167.48	0.91
Number of passenger cars emission group euro 2	1481.89	0.86	758.39	0.90	1519.40	0.85	752.10	0.91
Number of passenger cars emission group euro 3	1769.34	0.87	781.02	0.93	1785.75	0.86	1033.32	0.87
Number of passenger cars emission group euro 4	7027.27	0.88	3294.12	0.92	6786.87	0.89	3687.20	0.90
Number of passenger cars emission group euro 5	6157.90	0.91	3386.32	0.92	5917.91	0.91	3050.37	0.93
Number of passenger cars emission group euro 6r	6978.70	0.90	3579.00	0.92	6967.55	0.89	3269.41	0.94
Number of passenger cars emission group euro 6dt	4320.35	0.85	2555.25	0.87	4317.94	0.85	1461.44	0.95
Number of passenger cars emission group euro 6d	7072.20	0.70	8294.98	0.52	7472.18	0.59	8127.21	0.54
Number of passenger cars emission group euro other	2113.87	0.78	1627.34	0.78	2164.24	0.76	1289.55	0.86
Residential building living area	3.26	0.89	1.03	0.96	3.37	0.88	1.78	0.89
Non-residential building living area	0.09	0.83	0.06	0.91	0.08	0.84	0.07	0.88

To ensure transparency regarding the quality of the generated values in this work, a five-level confidence schema is introduced: *VERY HIGH*, *HIGH*, *MEDIUM*, *LOW*, and *VERY LOW*. This qualitative labeling system facilitates easier interpretation of data quality compared to conventional statistical measures such as RMSE or R-squared values.

Confidence assignment begins at the data collection stage: all non-missing values are automatically labeled as *VERY HIGH*. Missing values, in contrast, are assigned one of the remaining confidence levels based on the R-squared score of the chosen imputation model. Specifically, the thresholds are defined as follows: *HIGH* for >0.8, *MEDIUM* for >0.5 and ≤0.8, *LOW* for >0.2 and ≤0.5, and *VERY LOW* for ≤0.2.

For each variable, between the two experimental settings that are considered —with correlation thresholds of ≥0.1 and ≥0.5 —the configuration yielding the higher R-squared score is selected for imputing missing values. Although the XGBoost model is trained to minimize RMSE, R-squared scores are employed for quality ratings due to their bounded range (≤1), which provides a consistent and interpretable scale across variables. The final model selected for each variable, along with its corresponding confidence level, is presented in Table 8.

**Table 8:** The value confidence levels assigned based on the R-squared score obtained for the better-performing model between two predictor sets: those with a correlation threshold ≥0.1 and those with a correlation threshold ≥0.5.

Variable	Value confidence level	
	For correlation threshold ≥ 0.1	For correlation threshold ≥ 0.5
Utilized agricultural area	*HIGH*	—
Number of commerical and service companies	*MEDIUM*	—
Average daily traffic - light duty vehicles	*VERY LOW*	—
Employment in textile and leather manufacturing	—	*VERY LOW*
Employment in food and beverage manufacturing	*LOW*	—
Employment in mechanical and automotive engineering	—	*HIGH*
Employment in mechatronics, energy and electrical	—	*HIGH*
Employment in wood processing	*MEDIUM*	—
Number of passenger cars emission group euro 1	*HIGH*	—
Number of passenger cars emission group euro 2	—	*HIGH*
Number of passenger cars emission group euro 3	*HIGH*	—
Number of passenger cars emission group euro 4	*HIGH*	—
Number of passenger cars emission group euro 5	—	*HIGH*
Number of passenger cars emission group euro 6r	—	*HIGH*
Number of passenger cars emission group euro 6dt	—	*HIGH*
Number of passenger cars emission group euro 6d	—	*MEDIUM*
Number of passenger cars emission group euro other	—	*HIGH*
Residential building living area	*HIGH*	—
Non-residential building living area	*HIGH*	—

The corresponding confidence levels for R-squared thresholds are: *HIGH* for  >0.8, *MEDIUM* for  >0.5 and ≤0.8, *LOW* for  >0.2 and ≤0.5, and *VERY LOW* for ≤0.2.

The trained XGBoost models effectively predict missing values for most variables (see Table 8). However, the variables “employment in the food and beverage manufacturing sector” have *LOW* prediction quality, and “employment in textile and leather manufacturing” is classified as *VERY LOW*. The poor predictions for food and beverage manufacturing can be attributed to the lack of relevant predictor data at the NUTS3 level. In addition to this limitation, the low prediction quality for textile and leather manufacturing is further exacerbated by a higher proportion of missing values, with 34 out of 401 records missing, compared to other datasets at the NUTS3 level. The variable “Average daily traffic - light duty vehicles” shows the poorest prediction results. The R-squared values are negative (see Table 7), indicating that none of the predictors contribute meaningfully to the predictions. Out of 52 records, 10 have missing values. Reserving 10% of the remaining data for validation further reduces the number of records available for training a reliable XGBoost model. Therefore, the XGBoost predictions are discarded, and missing values are imputed using the mean of the existing data. Since this approach is not robust, the imputed values are assigned a *LOW* prediction quality.

**Evaluation of missing value prediction across countries**. As previously mentioned, missing values are observed only in the country-level datasets for Germany or Spain. Consequently, the validation using the 10% of data set aside specifically assesses how well missing values can be imputed within regions of the same country. Here, the trained models are applied to predict data for regions in the other country, and the results are analyzed.

**Fig. 11:**
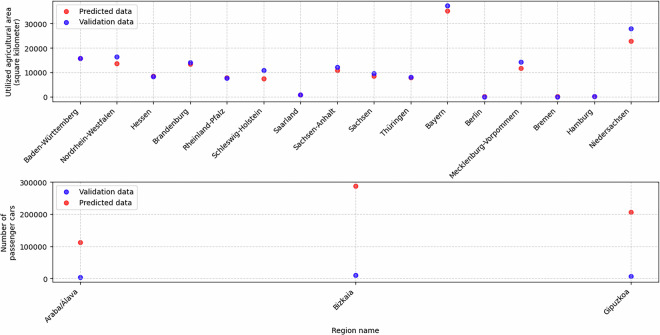
[Top] Results of training an XGBoost model to predict utilized agricultural area in Spain at the LAU level, and applying this model to estimate values for German LAU regions. The predicted data is compared with the available utilized agricultural area data at the NUTS1 level in Germany. The results indicate that the model’s predictions closely align with the actual data, with minimum and maximum deviations of 9.34 and 5106.56 square kilometers, respectively. [Bottom] Results of training an XGBoost model to predict passenger car stock in Germany at the NUTS3 level, and applying this model to estimate values for Spanish NUTS3 regions. The predicted data is compared with the available data for the 3 NUTS3 regions in the Basque Country, Spain. The results indicate that the model’s predictions deviate significantly from the actual data, with minimum and maximum deviations of 108957.0 and 276023.0 cars, respectively.

For instance, “utilized agricultural area” is available at the LAU level for Spain. A trained model, initially developed to impute missing values, is also applied to predict values for German LAU regions. These predictions are then validated against data from the Federal Statistical Office of Germany^24^, which provides agricultural area figures only at the NUTS1 level. To enable comparison, the predicted values are aggregated accordingly. Figure 11 demonstrates a strong alignment between the predictions and the validation data. This suggests that agricultural land use patterns in Spain and Germany are similar. The distribution of utilized agricultural area in both countries is well explained by highly correlated predictors such as total available area and non-irrigated arable land cover (Fig. 6). Given this successful validation, the XGBoost model is used to impute missing data for German LAU regions.

A similar validation is conducted at the NUTS3 level, where the number of passenger cars per emission group is estimated for the Spanish NUTS3 regions. The data is then aggregated into a single dataset representing the total number of passenger cars per NUTS3 region. A further aggregation to the NUTS2 level is performed, for comparison with data from Eustat^25^, which provides the number of cars for the three provinces of the Basque Country. Figure 11 presents this comparison, revealing significant deviations between the predictions and the validation data. A similar pattern is observed in other datasets at the German NUTS3 level. These discrepancies suggest that cross-country imputation is not universally reliable, potentially depending on the spatial level of analysis. The availability of more data at the LAU level provides greater variance, allowing for improved learning, whereas similar attempts at the NUTS3 level yield less accurate results. Additionally, sector-specific factors influence the effectiveness of imputation. For example, agricultural indicators exhibit similar spatial distributions in both countries, making cross-country imputation more feasible, whereas transport-related indicators do not follow the same pattern. Due to these inconsistencies, the predictions are discarded.

### Step-wise spatial disaggregation

Figure 2 shows that only some proxy data is readily available at the LAU level, while most statistical data is typically available at NUTS3 or NUTS2 levels. Additionally, most proxy datasets do not cover the Canary Islands in Spain. Due to this limitation, these regions are excluded from the scope of this study.

In this work, we perform a step-wise spatial disaggregation. Initially, proxy data available at the NUTS3 level is disaggregated using LAU proxy data, to achieve finer resolution. Subsequently, NUTS2 proxy data is disaggregated to the LAU level using both the LAU data and the previously disaggregated NUTS3 data as proxies. Finally, the emissions and FEC data are disaggregated to the LAU level. This approach improves the accuracy of the disaggregated data by progressively refining estimates.

Among the various spatial disaggregation approaches found in the literature, proxy data-based and machine learning-based methods are the most suitable for disaggregating emissions and FEC data^7^. The proxy data-based approach distributes the target data based on the proportion of the chosen spatial proxy. In contrast, the machine learning-based approach trains a predictive model, such as XGBoost, to learn the relationships between all available proxy data and the target data at the source spatial level (e.g., NUTS0), and then uses this model to predict the target values in each target region.

Initially, a machine learning-based approach for disaggregation was considered. The approach was eventually discarded due to the following reasons:

- The imputation of missing values resulted in poor predictions in certain cases. Applying an additional layer of prediction on top of this may further degrade the results.
- In Spain, there are only 52 NUTS3 regions, which may constitute a sample size too small to generate reliable predictions at the LAU level.
- Some variable pairs, such as population and gross domestic product, exhibited strong correlations at the NUTS3 level but weaker correlations at the LAU level. These differences in correlation raise concerns about whether the statistical relationships among variables, upon which the predictions are based, remain valid at the LAU level.
- For most variables, no validation data is available at the LAU level, making it challenging to assess the performance of this disaggregation approach.

Therefore, in this study, a proxy data-based spatial disaggregation method is employed. Here, the quality of disaggregated data primarily depends on how effectively the chosen proxy captures the spatial distribution of the target data. The selection of a spatial proxy is inherently constrained by the availability of data at fine-scale resolution. For each target dataset, potential proxies are initially identified based on theoretical considerations. If the most suitable proxy is unavailable in open databases with sufficient non-missing values, the closest alternative is selected. For example, in disaggregating employment data for textile and leather manufacturing, the ideal proxy would be the total size of textile and leather manufacturing facilities in each LAU region. If that data is unavailable, the next best option would be the number of such facilities, followed by a broader proxy such as “industrial or commercial units cover.” Since no data on textile and leather manufacturing facilities is accessible, “industrial or commercial units cover” is ultimately chosen as the proxy.

To provide transparency regarding the reliability of the disaggregated data, each proxy is assigned a confidence level —classified as *HIGH*, *MEDIUM*, *LOW*, or *VERY LOW* —to indicate its relevance and explanatory strength with respect to the target data. The confidence level reflects the degree of alignment between the proxy and the target dataset. For instance, in the example above, the total size of textile and leather manufacturing facilities would receive a *HIGH* confidence rating, the number of such facilities would receive a *MEDIUM* rating, and “industrial or commercial units cover” would be rated as *LOW*.

The confidence level assigned to the final disaggregated values at the LAU level is determined by taking the minimum of the confidence level of the proxy data and that of the proxy assignment. For instance, if a proxy value in a LAU region is of *MEDIUM* confidence, influenced by the missing value imputation, and the proxy assignment is of *LOW* confidence, then the disaggregated value will be assigned a *LOW* confidence. The selection of proxies for the step-wise spatial disaggregation process is outlined in the following.

**1. NUTS3 variables to LAU**. Tables 9, 10, and 11 list the NUTS3 variables together with their potential proxies. For each variable, the most suitable proxy is first identified. If this proxy is available in any public database, it is selected and assigned a *HIGH* confidence level. If the most suitable proxy is unavailable, the next best alternative is considered, assigning a *MEDIUM* confidence level, and the process is repeated as necessary until a suitable proxy dataset is obtained for disaggregation.

**Table 9:** The potential proxies for disaggregating each NUTS3 variable, commonly collected for both Germany and Spain.

NUTS3 Variable	Potential proxies		
	Most suitable	Alternative proxy 1	Alternative proxy 2
Employment in manufacturing	Industrial or commercial units cover + Population	—	—
Employment in construction	Number of construction companies	Construction sites cover + Road network	—
Road transport of freight	Number of heavy duty vehicles	Number of fuel stations	Road network
Heating degree days	Temperature	(No proxy. Same value for all child regions)	—
Number of cattle	Size of cattle farms	Number of cattle farms	Utilized agricultural area
Number of pigs	Size of pig farms	Number of pig farms	Utilized agricultural area
Number of buffaloes	Size of buffalo farms	Number of buffalo farms	Utilized agricultural area
Employment in agriculture, forestry, and fishing	Utilized agricultural area + Agroforestry areas cover + Number of fishers	Utilized agricultural area + Agroforestry areas cover + Water bodies cover + Water courses cover	—

The confidence level for the final selected proxy reflects the strength of its relationship with the target variable: *HIGH* if it is the most suitable, *MEDIUM* if it is the first alternative, and so forth.

**Table 10:** The potential proxies for disaggregating each NUTS3 variable, collected only for Germany.

NUTS3 Variable	Potential proxies		
	Most suitable	Alternative proxy 1	Alternative proxy 2
Employment in textile and leather manufacturing	Size of textile and leather manufacturing industries	Number of textile and leather manufacturing industries	Industrial or commercial units cover + Population
Employment in food and beverage manufacturing	Size of food and beverage manufacturing industries	Number of food and beverage manufacturing industries	ndustrial or commercial units cover + Population
Employment in mechanical and automotive engineering	Size of mechanical and automotive engineering industries	Number of mechanical and automotive engineering industries	Industrial or commercial units cover + Population
Employment in mechatronics, energy and electrical	Size of mechatronics, energy and electrical industries	Number of mechatronics, energy and electrical industries	Industrial or commercial units cover + Population
Employment in wood processing	Size of wood processing industries	Number of wood processing industries	Industrial or commercial units cover + Population
Number of passenger cars emission group euro 1	Age distribution of passenger cars	Number of fuel stations	Population
Number of passenger cars emission group euro 2	Age distribution of passenger cars	Number of fuel stations	Population
Number of passenger cars emission group euro 3	Age distribution of passenger cars	Number of fuel stations	Population
Number of passenger cars emission group euro 4	Age distribution of passenger cars	Number of fuel stations	Population
Number of passenger cars emission group euro 5	Age distribution of passenger cars	Number of fuel stations	Population
Number of passenger cars emission group euro 6r	Age distribution of passenger cars	Number of fuel stations	Population
Number of passenger cars emission group euro 6dt	Age distribution of passenger cars	Number of fuel stations	Population
Number of passenger cars emission group euro 6d	Age distribution of passenger cars	Number of fuel stations	Population
Number of passenger cars emission group euro other	Age distribution of passenger cars	Number of fuel stations	Population
Residential building living area	Population	—	—
Non-residential building living area	Industrial or commercial units cover + Population	—	—

The confidence level for the final selected proxy reflects the strength of its relationship with the target variable: *HIGH* if it is the most suitable, *MEDIUM* if it is the first alternative, and so forth.

**Table 11:** The potential proxies for disaggregating each NUTS3 variable, collected only for Spain.

NUTS3 Variable	Potential proxies		
	Most suitable	Alternative proxy 1	Alternative proxy 2
Number of commerical and service companies	Industrial or commercial units cover + Population	—	—
Average daily traffic - light duty vehicles	Number of light duty vehicles	Number of fuel stations	Population

The confidence level for the final selected proxy reflects the strength of its relationship with the target variable: *HIGH* if it is the most suitable, *MEDIUM* if it is the first alternative, and so forth.

It is important to note that some proxies are added although they have different measurement units. For example “construction sites cover” and “road network” are expressed in square kilometer and kilometer. To ensure comparability, all variables are first normalized by their maximum values, preserving true zeros while scaling all other values relative to the highest observed value. This normalization allows proxies to be summed without introducing inconsistencies.

The “industrial or commercial units cover” data is sourced from the Corine Land Cover database, which includes only industrial and commercial units spanning 25 hectares or more. Due to this threshold, many regions have zero values. Since this limitation is consistent across all regions, data imputation was not feasible. Consequently, this proxy is used in conjunction with population data in this study. Furthermore, “employment in construction” uses “construction sites cover” and “road network” as proxies because construction encompasses both building and road construction.

**2. NUTS2 variables to LAU**. Table 12 details the proxy assignment process in the case of the NUTS2 variables “number of motorcycles”, “air transport of passengers”, and “air transport of freight”.

**Table 12:** The potential proxies for disaggregating each NUTS2 variable, commonly collected for both Germany and Spain.

NUTS2 Variable	Potential proxies		
	Most suitable	Alternative proxy 1	Alternative proxy 2
Number of motorcycles	Number of motorcycle service stations	Number of fuel stations	Road network
Air transport of passengers	Airports cover	—	—
Air transport of freight	Airports cover	—	—

The confidence level for the final selected proxy reflects the strength of its relationship with the target variable: *HIGH* if it is the most suitable, *MEDIUM* if it is the first alternative, and so forth.

**3b. FEC (NUTS0 data) to LAU**. Table 13 presents the FEC end-use sectors for which final proxies were available in both countries. Table 14 lists the FEC end-use sectors for which final proxies were available exclusively for Germany. Here, emissions from passenger car road transport are disaggregated according to the passenger car fleet categorized into different emission groups. These groups define the vehicle emission standards used in Europe^26^, with each group setting caps on specific air pollutants. The initial emission group, Euro 1, was introduced in July 1992. Over the years, the standards have become increasingly stringent with the introduction of new emission caps. The caps for diesel passenger cars concerning pollutants such as carbon monoxide (*CO*), hydrocarbons and nitrogen oxides (*HC* + *NO*_*X*_), and particulate matter (*PM*) are detailed in Table 15. For each emission group, the caps for these three pollutants are summed to obtain a weighting factor for the proxies. The passenger car data provides information for emission group 5 but does not differentiate between Euro 5a and 5b. In this case, the more lenient tier, Euro 5a, is considered to assign more emissions to cars in tier 5. The data also includes an emission group labeled “Other.” Due to the lack of additional information from the data source regarding this category, it is treated as the Euro 1 group. Since this data was unavailable for Spain, “average daily traffic - light duty vehicles” is used as a proxy. Similarly, Table 16 outlines the FEC end-use sectors for which final proxies were available for Spain.

**Table 13:** FEC end-use sectors with final proxies commonly available for both Germany and Spain.

Emission source	Potential proxies	
	Most suitable	Alternative proxy 1
Iron and steel industries	Capacity of iron and steel industries	Number of iron and steel industries
Non-ferrous metals industries	Capacity of non-ferrous metals industries	Number of non-ferrous metals industries
Chemical industries	Capacity of chemical industries	Number of chemical industries
Non-metallic minerals industries	Capacity of non-metallic minerals industries	Number of non-metallic minerals industries
Mining and quarrying	Mineral extraction sites cover	—
Paper, pulp, and printing industries	Capacity of paper, pulp, and printing industries	Number of paper and printing industries
Construction	Employment in construction	—
Rail transport	Railway network	—
Domestic aviation	Air transport of freight + Air transport of passengers	—
Domestic navigation	Port areas cover	—
Agriculture and forestry	Employment in agriculture, forestry, and fishing	—

The confidence level for the final selected proxy reflects the strength of its relationship with the target variable: *HIGH* if it is the most suitable, *MEDIUM* if it is the first alternative, and so forth.

**Table 14:** FEC end-use sectors with final proxies available for Germany.

FEC source	Most suitable proxy
Wood and wood products industries	Employment in wood processing
Transport equipment industries	Employment in mechanical and automotive engineering
Machinery industries	Employment in mechatronics, energy and electrical
Food, beverages, and tobacco industries	Employment in food and beverage manufacturing
Textile and leather industries	Employment in textile and leather manufacturing
Road transport	(Road transport of freight) + (3.83 * Number of passenger cars emission group euro 1) + (1.78 * Number of passenger cars emission group euro 2) + (1.25 * Number of passenger cars emission group euro 3) + (0.825 * Number of passenger cars emission group euro 4) + (0.735 * Number of passenger cars emission group euro 5) + (0.6745 * Number of passenger cars emission group euro 6r) + (0.6745 * Number of passenger cars emission group euro 6dt) + (0.6745 * Number of passenger cars emission group euro 6d) + (3.83 * Number of passenger cars emission group euro other)
Households	Residential building living area * Heating degree days
Commerce	Non-residential building living area * Heating degree days

The confidence level for the final selected proxy reflects the strength of its relationship with the target variable: *HIGH* if it is the most suitable, *MEDIUM* if it is the first alternative, and so forth.

**Table 15:** Emission caps for different air pollutants, per emission group.

Tier	CO	HC + NO_X_	PM	Total
Euro 1	2.72	0.97	0.14	3.83
Euro 2	1.0	0.7	0.08	1.78
Euro 3	0.66	0.56	0.05	1.25
Euro 4	0.50	0.30	0.025	0.825
Euro 5a	0.50	0.230	0.005	0.735
Euro 5b	0.50	0.230	0.0045	0.7345
Euro 6b	0.50	0.170	0.0045	0.6745
Euro 6c	0.50	0.170	0.0045	0.6745
Euro 6d-temp	0.50	0.170	0.0045	0.6745
Euro 6d	0.50	0.170	0.0045	0.6745
Euro 6e	0.50	0.170	0.0045	0.6745

**Table 16:** FEC end-use sectors with final proxies available for Spain.

FEC source	Potential proxies	
	Most suitable	Alternative proxy 1
Wood and wood products industries	Employment in wood processing	Employment in manufacturing
Transport equipment industries	Employment in mechanical and automotive engineering	Employment in manufacturing
Machinery industries	Employment in mechatronics, energy and electrical	Employment in manufacturing
Food, beverages, and tobacco industries	Employment in food and beverage manufacturing	Employment in manufacturing
Textile and leather industries	Employment in textile and leather manufacturing	Employment in manufacturing
Road transport	Road transport of freight + Average daily traffic - light duty vehicles	—
Households	Residential building living area * Heating degree days	Population * Heating degree days
Commerce	Non-residential building living area * Heating degree days	Number of commercial and service companies * Heating degree days

The confidence level for the final selected proxy reflects the strength of its relationship with the target variable: *HIGH* if it is the most suitable, *MEDIUM* if it is the first alternative, and so forth.

**3a. GHG emissions (NUTS0 data) to LAU**. Tables 17, 18, and 19 present the proxy assignments in the case of emissions end-use sectors. The proxies are similar to those used for FEC. The differences arise from a different breakdown of the source sub-sectors.

**Table 17:** GHG emissions end-use sectors with final proxies commonly available for both Germany and Spain.

Emission source	Potential proxies	
	Most suitable	Alternative proxy 1
Iron and steel industries	Capacity of iron and steel industries	Number of iron and steel industries
Non-ferrous metals industries	Capacity of non-ferrous metals industries	Number of non-ferrous metals industries
Chemical industries	Capacity of chemical industries	Number of chemical industries
Non-metallic minerals industries	Capacity of non-metallic minerals industries	Number of non-metallic minerals industries
Paper, pulp, and printing industries	Capacity of paper, pulp, and printing industries	Number of paper and printing industries
Rail transport	Railway network	—
Road freight transport	Road transport of freight	—
Road transport using motorcycles	Number of motorcycles	—
Domestic aviation	Air transport of freight + Air transport of passengers	—
Domestic navigation	Port areas cover	—
Cultivation	Utilized agricultural area	—
Livestock	Number of cattle + Number of pigs + Number of buffaloes	—

The confidence level for the final selected proxy reflects the strength of its relationship with the target variable: *HIGH* if it is the most suitable, *MEDIUM* if it is the first alternative, and so forth.

In Germany, except for energy-intensive industries, all proxies correspond directly to relevant emission sources. As a result, most emissions end-use sector proxies are classified as having *HIGH* confidence (see Table 18). In contrast, Spain lacks detailed employment data and spatial data on residential and non-residential areas, limiting the availability of *HIGH* confidence proxies for several emissions end-use sectors (see Table 19).

**Table 18:** GHG emissions end-use sectors with final proxies available for Germany.

Emission source	Most suitable proxy
Food, beverages, and tobacco industries	Employment in food and beverage manufacturing
Other manufacturing industries and construction	Employment in mechanical and automotive engineering + Employment in mechatronics, energy and electrical + Employment in textile and leather manufacturing + Employment in construction
Road transport using cars	(3.83 * Number of passenger cars emission group euro 1) + (1.78 * Number of passenger cars emission group euro 2) + (1.25 * Number of passenger cars emission group euro 3) + (0.825 * Number of passenger cars emission group euro 4) + (0.735 * Number of passenger cars emission group euro 5) + (0.6745 * Number of passenger cars emission group euro 6r) + (0.6745 * Number of passenger cars emission group euro 6dt) + (0.6745 * Number of passenger cars emission group euro 6d) + (3.83 * Number of passenger cars emission group euro other)
Households	Residential building living area * Heating degree days
Commerce	Non-residential building living area * Heating degree days

The confidence level for the final selected proxy reflects the strength of its relationship with the target variable: *HIGH* if it is the most suitable, *MEDIUM* if it is the first alternative, and so forth.

**Table 19:** GHG emissions end-use sectors with final proxies available for Spain.

Emission source	Potential proxies	
	Most suitable	Alternative proxy 1
Food, beverages, and tobacco industries	Employment in food and beverage manufacturing	Employment in manufacturing
Other manufacturing industries and construction	Employment in mechanical and automotive engineering + Employment in mechatronics, energy and electrical + Employment in textile and leather manufacturing + Employment in construction	Employment in manufacturing + Employment in construction
Road transport using cars	Average daily traffic - light duty vehicles	—
Households	Residential building living area * Heating degree days	Population * Heating degree days
Commerce	Non-residential building living area * Heating degree days	Number of commerical and service companies * Heating degree days

The confidence level for the final selected proxy reflects the strength of its relationship with the target variable: *HIGH* if it is the most suitable, *MEDIUM* if it is the first alternative, and so forth.

### Data validation

Finally, the spatial disaggregation results are compared with the values reported in local inventories (NetZeroCities^4^) and an open-source sub-national emissions dataset (EDGAR^8^), ensuring alignment with the sub-sectors considered in this study. The details of this validation process are presented in the technical validation section of this manuscript.

## Data Record

The generated datasets offer sectorally-detailed municipal/LAU level FEC and GHG emissions data, for Germany and Spain, for the year 2022^27^. The datasets are available on Zenodo at https://zenodo.org/records/14097217. There are two sub-folders in this repository, one for the FEC data and the other for the emissions data. Within each sub-folder, several .csv files exist. Each .csv file provides data for one sub-sector.

The .csv files contain “region_code”, “value”, and “value_confidence_level” columns. The “region_code” is in the format  < *NUTS*3 > _ < *LAU* > i.e., the NUTS3 code is prepended with an “_” to the LAU code, to identify parent NUTS3 regions for each LAU region. The “value” column provides the values in the region. The FEC values are expressed in MWh and the emissions values are expressed in Mt. The “value_confidence_level” is an aggregate of the confidence in spatial proxy missing value imputation and the confidence in the appropriateness of the spatial proxy.

The LAU codes are taken from Eurostat for the year 2019 and the parent NUTS3 codes are for the year 2016. These can be accesed on Eurostat under https://ec.europa.eu/eurostat/web/gisco/geodata/statistical-units/local-administrative-units.

The Zenodo repository also includes a readme file detailing this information.

## Technical Validation

This study introduces a spatial disaggregation workflow that requires technical validation at two critical stages: (1) the imputation of missing values in proxy data, and (2) the final disaggregation of FEC and emissions data. The validation of the missing value imputation was shown already in the Methods section.

Validating the disaggregated data is more challenging due to the absence of data on energy consumption and emissions at municipal/LAU level in official databases. This lack of data is the primary reason for performing spatial disaggregation of national data. Here, technical validation is carried out through the following approaches:

- **City-level inventories:** Bottom-up inventories reported by selected Spanish cities are used for validation.
- **Cross-validation with disaggregated product:** The results are evaluated against another spatially disaggregated dataset, namely EDGAR. While such datasets are useful for comparative analysis, none provide comprehensive coverage of all the sub-sectors addressed in this study at the municipal level. Furthermore, these alternative datasets are themselves the outcome of spatial disaggregation processes rather than being grounded in official statistics, and therefore warrant independent critical assessment.
- **Visual assessment:** For sectors lacking direct reference datasets, visual inspections are performed to confirm that the spatial distribution of emissions aligns with the patterns of the proxy data used.

The results are discussed in the following sub-sections.

### Validation against city-level inventories

We compare the disaggregated results with the FEC and emissions reported by seven Spanish cities —Barcelona, Madrid, Valencia, Valladolid, Vitoria-Gasteiz, Zaragoza, and Seville —as part of the Climate-Neutral and Smart Cities initiative^28^. The climate action plans developed by these cities align in terms of baseline year (2019) and sectoral coverage^4^. Accordingly, we used 2019 national values for disaggregation to ensure comparability with the reported bottom-up inventories for matching end-use sectors. Two sectors —buildings and road transport —could be aligned across datasets, and comparisons were therefore limited to these sectors.

**Fig. 12:**
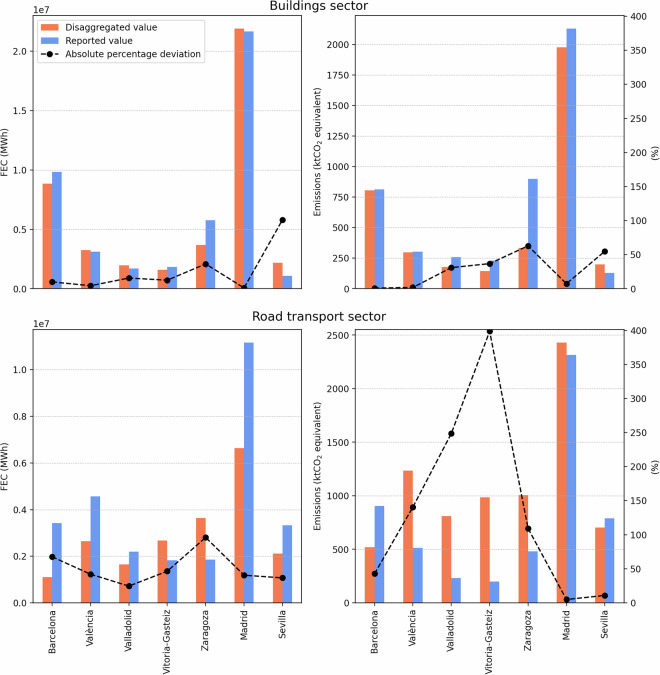
Comparison of reported and disaggregated FEC [left] and emissions [right] across selected Spanish municipalities, for the buildings sector [top] and road transport sector [bottom]. In each subplot, bars represent the reported and disaggregated values (left y-axis), while the black line indicates the absolute percentage deviation between them (right y-axis). The figure highlights deviations between top-down disaggregated estimates and values reported in local inventories. Notable discrepancies in Zaragoza and Seville are examined in detail, illustrating the influence of differences in spatial reporting levels and sectoral definitions. Emissions deviations underscore the impact of regional variations in energy mix, while inconsistencies in transport sector reporting contribute to further disparities.

Figure 12 presents a comparison of the reported and disaggregated values. For the building sector, which includes both household and commerce sectors, in most cases, the absolute deviation in FEC values remains below 20%, with notable exceptions in Zaragoza and Seville.

In the case of Zaragoza, further investigation revealed that the reported FEC corresponds to NUTS3 level rather than the LAU level. This is confirmed by the municipality’s SECAP^29^, where the building sector FEC is reported as 3,664,235 MWh. Our disaggregated estimate for Zaragoza municipality is 3,670,931 MWh —resulting in a deviation of only 0.18%. When disaggregated values are summed to represent the entire NUTS3 region, the resulting FEC is 6,187,351 MWh, which deviates by just 7.26% from the reported value.

In Seville, the discrepancy arises because only residential electricity consumption is reported under the building sector, excluding other significant components such as commercial and non-electrical energy consumption. This leads to a larger deviation from the disaggregated estimate. These findings highlight the value of top-down disaggregation approaches as a complementary tool to bottom-up inventories, particularly in identifying inconsistencies or omissions in local reporting.

Figure 12 also presents a comparison of emission values. Although the same proxies were used for disaggregating both FEC and emissions, the disaggregated emission figures show greater deviation from those reported in bottom-up inventories compared to FEC values. This discrepancy can be attributed to differences in the energy mix between national and regional levels. For instance, according to Eurostat’s national energy balance data, the commerce sector uses 19.16% natural gas. In contrast, the share of natural gas usage in the commerce sector in the provinces of Araba, Bizkaia, and Gipuzkoa is 26.32%, 24.38%, and 19.71%, respectively^30^. These variations in energy mix can significantly impact emission estimates. Therefore, when developing local inventories using top-down datasets, it is crucial to recalculate emissions if the regional energy mix diverges notably from the national average.

The local inventories also include data for the road transport sector; however, the reporting practices for this sector are often unclear and inconsistent. For instance, in Vitoria-Gasteiz, the reported FEC appears to cover only road transport, whereas in Barcelona, railway transport is also included. In contrast, Valencia’s plan explicitly states that only road transport within the city limits is considered. These inconsistencies in sectoral definitions and reporting scope likely contribute to the significant deviations observed in Fig. 12.

### Cross-validation with EDGAR

EDGAR provides disaggregated emissions data by sector at the NUTS2 level for the year 2022. As a first step, we conduct a sectoral comparison to identify categories that align between datasets. Table 20 lists the sectors identified as comparable, along with the corresponding national totals reported by both EDGAR and Eurostat.

**Table 20:** Comparison of sectoral values reported by EDGAR database and Eurostat values at NUTS0 level, to determine matching sectors.

Sector	Germany			Spain		
	EDGAR value	Eurostat value	Percentage deviation	EDGAR value	Eurostat value	Percentage deviation
Industry	188.81	115.80	38.67	81.24	37.86	53.40
Buildings	128.05	109.69	14.34	36.88	24.32	34.06
Transport	143.38	147.27	−2.71	83.51	90.21	−8.02
Agriculture	55.81	51.72	7,33	44.46	34.86	21.59

Note: The unit of measure of the values is kt CO2 equivalent.

With the exception of the transport sector, all categories exhibit absolute deviations exceeding 20%. These discrepancies may arise from differences in the inclusion or exclusion of certain sub-sectors. For instance, chemical industry emissions are not reported for Germany in Eurostat, and thus are not considered here. Moreover, emissions from power plants appear to be included under the industrial sector in EDGAR, whereas they are excluded in our categorisation.

**Fig. 13:**
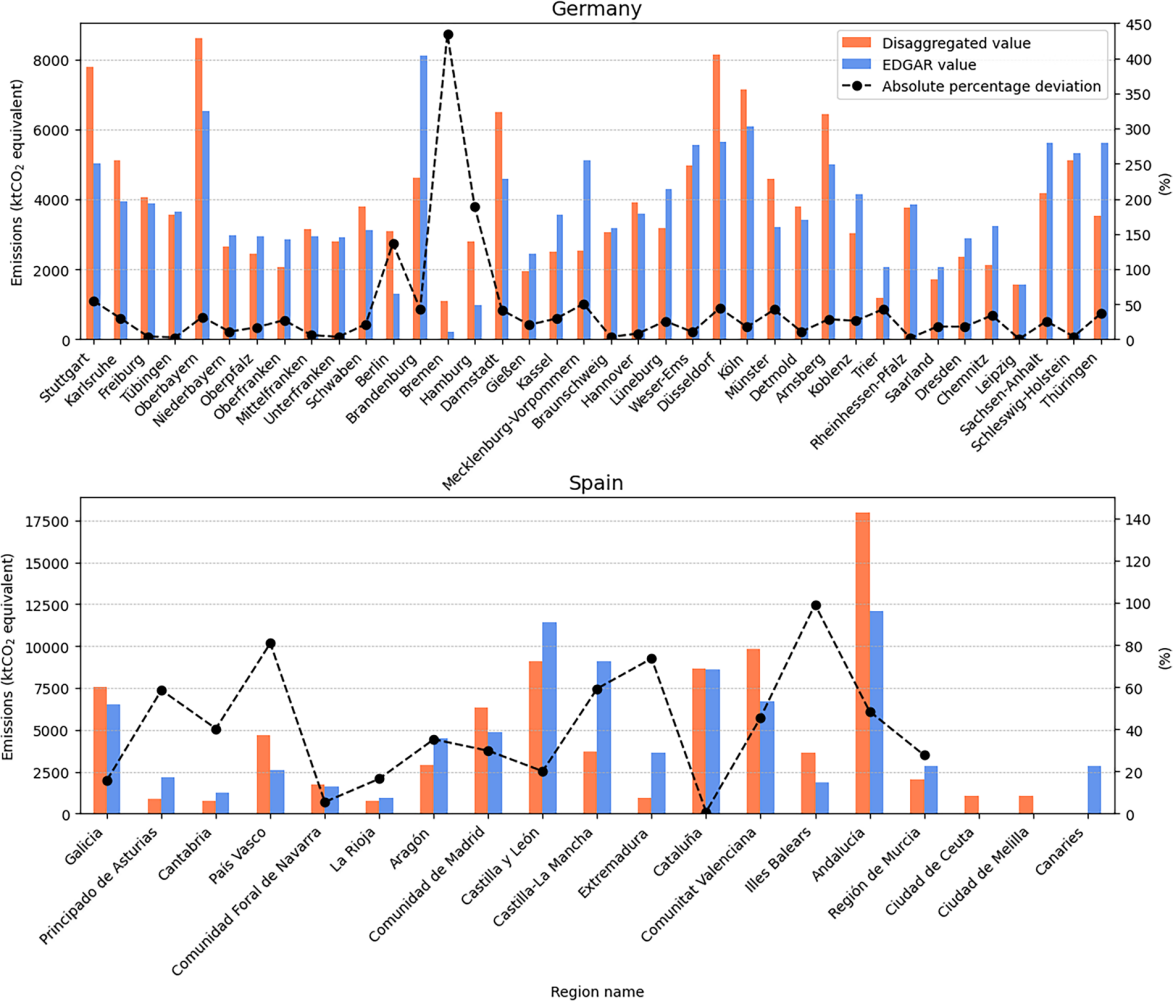
Comparison of aggregated transport emissions at the NUTS2 level between this study’s disaggregated dataset and the EDGAR database for Germany [top] and Spain [bottom]. Bars represent emissions values from both datasets (left y-axis), while the black line indicates the absolute percentage deviation between them (right y-axis). Despite close alignment at the national level, notable regional discrepancies emerge due to factors such as differences in regional coverage and spatial proxy selection in both works.

Given the relatively minor deviation observed in the transport sector at the national scale, a more granular comparison at the NUTS2 level was conducted. To facilitate this, emissions data from all LAU regions were aggregated to their corresponding NUTS2 regions. Figure 13 presents this comparison, alongside the associated percentage deviations.

Despite the low national-level discrepancies, significant regional variations are evident across both countries in the two datasets. These regional discrepancies can be attributed to several factors:

- *Propagation of national-level differences:* Although national totals exhibit only minor deviations, these discrepancies are distributed across regions, potentially amplifying inconsistencies at the sub-national level.
- *Differences in regional coverage:* In the case of Spain, the Canary Islands were excluded from our analysis due to the unavailability of suitable proxy datasets. Conversely, EDGAR includes this NUTS2 region but omits the autonomous cities of Ceuta and Melilla, which are accounted for in our dataset.
- *Choice and availability of spatial proxies:* Our approach leverages openly available regional-level datasets as proxies for spatial disaggregation. While EDGAR also employs open data sources, the specific proxies used are not always transparent, making it difficult to isolate the precise causes of spatial discrepancies between the datasets.

### Visual assessment for non-matching sub-sectors

**Fig. 14:**
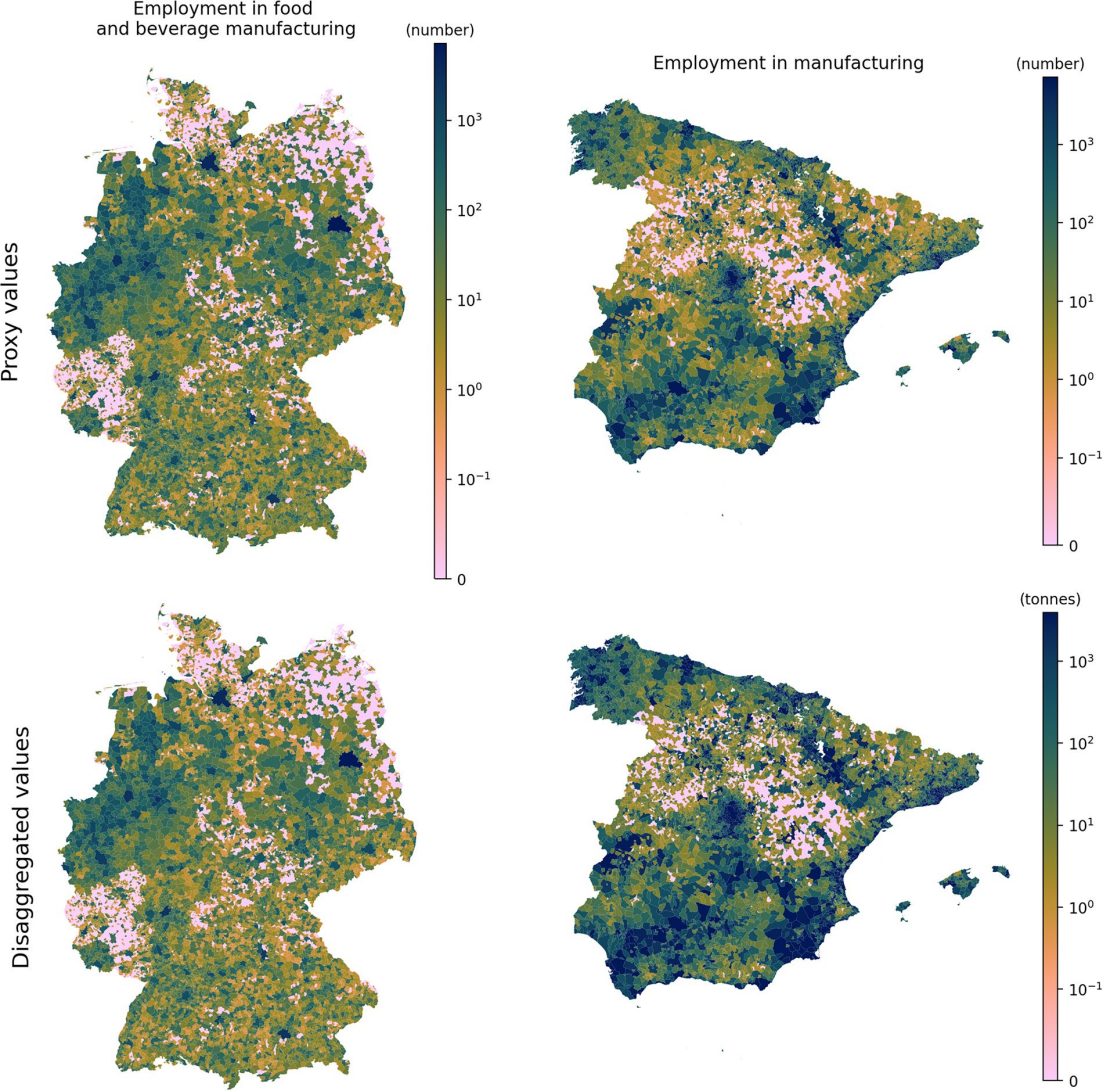
[top-left] “Employment in food and beverage manufacturing” for Germany [top-right] “Employment in manufacturing” in Spain. These proxies are used todisaggregate the emissions in food, beverages, and tobacco industries. [bottom] Disaggregated emission values.

Figure 14 illustrates the spatial distribution of the proxy data, namely “employment in food and beverage manufacturing” and “employment in manufacturing” for Germany and Spain, respectively. These proxies are used to disaggregate emissions in the food, beverages, and tobacco industries. The figure also displays the disaggregated emission values. It can be observed that the spatial distribution of the disaggregated values mirrors that of the proxy data, confirming that the disaggregation has been performed accurately. Figures pertaining to other sub-sectors are available on GitHub along with the code. The link can be found under the Code Availability section.

### Limitations and prospects

This study aimed to generate spatially and sectorally detailed energy consumption and emissions data for Germany and Spain using a proxy data-based spatial disaggregation approach built on publicly available datasets. The use of open data was intentional to ensure replicability and applicability in other national contexts. However, several limitations emerged that influence the accuracy and completeness of the results.

A key limitation lies in the availability and granularity of suitable proxy data. Sector-specific proxies at the municipal level were rarely available, necessitating a step-wise disaggregation approach —first from intermediate spatial units to municipal level, and subsequently national values to municipalities. Even with this method, suitable proxies could not be identified for all sectors in both countries. In particular, sectors such as textile manufacturing or light-duty vehicle traffic lacked strong, relevant proxy indicators, and thus generalized proxies were employed. This likely introduced spatial inaccuracies and contributed to variability in disaggregation accuracy across sectors and regions.

Additionally, several proxy datasets contained missing values. While XGBoost was used to impute these gaps effectively in most cases, its predictive performance was constrained for certain variables, primarily due to a lack of strong predictor variables. This further reduced confidence in the imputed values and, by extension, in the disaggregated estimates for affected sectors.

Validation of the results presents another limitation. Only a small set of city-level inventories from Spain and one external dataset were available for benchmarking. These validation sources were limited in both spatial coverage and sectoral completeness. In particular, the Spanish city inventories covered only seven municipalities and lacked representation for all sectors modeled in this study. No equivalent bottom-up inventories were available for German municipalities, precluding any robust cross-country validation. The external dataset, although covering both countries, is itself a product of spatial disaggregation and showed significant deviations from our results, further complicating the assessment of overall accuracy.

The study also captures only a single reference year (2022) and does not incorporate temporal dynamics or trends. This constrains the direct applicability of the results for time-sensitive planning or forecasting and underscores the static nature of the current dataset. Additionally, the disaggregation approach employed does not account for potential spatial autocorrelation within the data. Incorporating spatial autocorrelation-particularly relevant in sectors such as transport where cross-regional interactions are common-could further enhance the accuracy of the disaggregated results.

Given these constraints, the outputs of this study should be interpreted with caution, particularly in regions or sectors where data coverage is sparse or confidence scores are low. Nevertheless, as data availability improves, particularly at the municipal level, and as more reliable and comprehensive bottom-up inventories become accessible, there is significant scope to improve the accuracy and robustness of the results presented here. The integration of more targeted proxy indicators and a broader validation framework would enhance the credibility and utility of the datasets. Continued efforts in data harmonization, transparency, and open-access publication of sub-national inventories will be essential to support this advancement.

## Usage Notes

This study employed the 2019 LAU definitions. It is important to note that LAU boundaries can change annually. As such, future analyses using updated LAU definitions may require reconfiguration of the disaggregation process to ensure alignment with the revised regional boundaries. Additionally, any spatial proxy data collected at the LAU level would need to be reprocessed accordingly. At broader spatial scales, such as NUTS3, boundary definitions are typically updated on a four-year cycle. For this study, we used the 2016 NUTS definitions.

The choice of reference year for regional definitions, as well as the year of data collection, is critical, as both can significantly influence disaggregation outcomes. In this analysis, emissions and FEC data were collected for the year 2022. Proxy data, however, originate from various years, with preference given to the most recent data available from each source. For example, population data from 2019 was used in conjunction with the 2019 LAU definitions, while land cover information was derived from the Corine Land Cover dataset, last updated in 2018.

Therefore, if the disaggregation workflow presented here is applied using updated regional definitions and/or more recent datasets, the resulting outputs may differ from those reported in this study.

## Data Availability

The spatial disaggregation workflow developed for this work is implemented in Python and is available on GitHub under https://github.com/FZJ-IEK3-VSA/EnergyEmissionsRegio. The core functions can be accessed in the “energyemissionsregio” directory, while the sections on missing value imputation and disaggregation are in the “experiments” directory.
